# RNA-based cooperative protein labeling that permits direct monitoring of the intracellular concentration change of an endogenous protein

**DOI:** 10.1093/nar/gkab839

**Published:** 2021-09-28

**Authors:** Kathleen Beverly Alog Pe, Kenji Yatsuzuka, Hayase Hakariya, Tomoki Kida, Yousuke Katsuda, Masatora Fukuda, Shin-ichi Sato

**Affiliations:** Institute for Chemical Research, Kyoto University, Uji, Kyoto 611-0011, Japan; Institute for Chemical Research, Kyoto University, Uji, Kyoto 611-0011, Japan; Institute for Chemical Research, Kyoto University, Uji, Kyoto 611-0011, Japan; Division of Materials Science and Chemistry, Faculty of Advanced Science and Technology, Kumamoto University, 2-39-1 Kurokami, Chuo-ku, Kumamoto 860-8555, Japan; Institute for Integrated Cell-Material Sciences (WPI-iCeMS), Kyoto University, Kyoto 606-8501, Japan; Division of Materials Science and Chemistry, Faculty of Advanced Science and Technology, Kumamoto University, 2-39-1 Kurokami, Chuo-ku, Kumamoto 860-8555, Japan; Department of Chemistry, Faculty of Science, Fukuoka University, 8-19-1 Nanakuma, Jonan-ku, Fukuoka 814-0180, Japan; Institute for Chemical Research, Kyoto University, Uji, Kyoto 611-0011, Japan

## Abstract

Imaging the dynamics of proteins in living cells is a powerful means for understanding cellular functions at a deeper level. Here, we report a versatile method for spatiotemporal imaging of specific endogenous proteins in living mammalian cells. The method employs a bifunctional aptamer capable of selective protein recognition and fluorescent probe-binding, which is induced only when the aptamer specifically binds to its target protein. An aptamer for β-actin protein preferentially recognizes its monomer forms over filamentous forms, resulting in selective G-actin staining in both fixed and living cells. Through actin-drug treatment, the method permitted direct monitoring of the intracellular concentration change of endogenous G-actin. This protein-labeling method, which is highly selective and non-covalent, provides rich insights into the study of spatiotemporal protein dynamics in living cells.

## Graphical Abstract

**Graphical Abstract:**
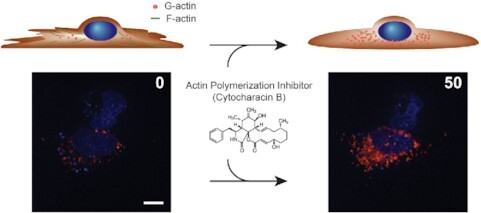
A convenient and highly-selective protein-labeling technology permitted direct monitoring of intracellular concentration change of endogenous G-actin proteins, which is normally very hard to distinguish from F-actin by live-cell staining.

## INTRODUCTION

Imaging of subcellular localization and dynamics of proteins is a powerful means for elucidating biochemical pathways in cells. Immunostaining, a method used with antibodies to visualize the intracellular distribution of protein in fixed cells, has an edge, but it provides only a snapshot image taken at the time of fixation. Therefore, establishing live-cell protein imaging methods has ever been in high demand, and a number of unique methods have been proposed to visualize protein dynamics in living cells. Out of the numerous techniques developed, the most widely used method to date is the overexpression of the protein of interest as a fusion with a fluorescent protein (1,2) or a split fluorescent protein (3–11). In more recently developed approaches, proteins of interest are fused to a small reactive peptide or protein such as the arsenic-based FLASH tag (12,13) and the PYP tag (14–16). These methods allowed visualization of protein dynamics in living cells, yet their use was limited to exogenously overexpressed proteins. Very recently, CRISPR/Cas9 genome editing technology has made it possible to knock in a fluorescent-protein-tagged protein of interest for visualizing its molecular dynamics in living cells or tissues (17). However, such modified or overexpressed proteins may not always reflect the behavior of the endogenous proteins in living cells (18,19).

To overcome such limitations, an unconventional live-cell imaging technology that is applicable to non-engineered, endogenous proteins would be useful to a notable extent. One of the most powerful approaches ever developed so far is ligand-directed labeling, where binding of a protein target to its modified ligand leads to proximity-induced covalent labeling of the protein. Selective protein labeling, such as using quenched ligand-directed tosylate compounds (20–23) and diazocoumarin compounds (24), permitted visualization of native endogenous proteins in cells. The approach does have some drawbacks; for example, the target protein must have a known ligand that can be a handle for fluorescent labeling. Although an aptamer-mediated indirect quantum dot labeling method (25) has enabled the observation of protein dynamics without ligand support, irreversible protein labeling, including ligand-directed methods, does not allow us to observe molecular dynamics with changes in protein concentration. Such drawbacks in irreversible protein labeling have been overcome by taking advantage of antibody-based peptides (26). For instance, a 17-amino-acid Lifeact peptide probe, which can specifically label F-actin without adverse effects on actin dynamics, has been reported (27,28). Fluorescently labeled nanobodies have also been developed to visualize endogenous protein dynamics (29). They may still be of limited utility due to their own background fluorescence, although the chemically modified peptides allow visualization of protein dynamics in living cells. Another powerful approach is the introduction of non-canonical amino acids, which can be modified with fluorescent dyes into the proteins of interest (30,31). Despite its usefulness, the general use of this approach has been hampered by the requirement of orthogonal aminoacyl tRNA synthetase/tRNA pairs (31,32). Thus, an easy-to-use live-cell imaging method for a specific endogenous protein would be a promising tool that accelerates the understanding of spatial and temporal protein dynamics in living mammalian cells. Herein we report a convenient and highly selective protein-labeling method that permits direct monitoring of the intracellular concentration change of an endogenous protein in human cells.

## MATERIALS AND METHODS

Additional details are available in the supplemental information.

### General procedures

ESI-mass spectra for the probes and peptides were measured with an LCMS-2020 system (Shimadzu). HPLC purification and profiling was done with an Inertsil ODS-3 reversed phase column (GL Sciences, 5 μm, 20 × 100 mm and 2.1 × 100 mm), with a linear gradient of 0.1% trifluoroacetic acid in H_2_O and 0.1% trifluoroacetic acid in acetonitrile (0−90%) as the solvent, and a flow rate of 3.0 ml/min. All fluorescence spectra were measured with an LS 55 fluorescence spectrometer (Perkin Elmer). Fluorescence spectra were recorded using a FluoroMax^®^-4 (HORIBA, Ltd.). The live-cell and fixed-cell imaging were performed using a CellVoyager™ CV1000 Confocal Scanner Box (Yokogawa Electric Corporation) equipped with an ultra-sensitive EMCCD C9100-13 camera (512 × 512 pixels) and an Olympus UPLSAPO 100XO 1.4 oil objective.

### *In vitro* selection procedure

The original double-stranded DNA pool was constructed via PCR, using a synthetic oligonucleotide containing 20 random nucleotides flanking both sides of the reported BHQ1 binding sequence [5′- GGA TCC AAG CTT GTT TGG C - N_20_ - TGG CCT AGA TAA ATT CGG AGC TT - N_20_ - GCT TTC GAC GGA GAA TTC -3′] as the template. All PCR reactions were done with KOD FX Neo (Toyobo). Primers used for PCR amplification were: forward [5′-GCT AAT ACG ACT CAC TAT AGG GAT CCA AGC TTG T-3′ (T7 promoter sequence is underlined)] and reverse [5′-GAA TTC TCC GTC GAA AG-3′]. The original DNA pool was *in vitro* transcribed with T7 RNA polymerase (T7 MegaScript Kit, Ambion) to give the first RNA pool. After transcription, the mixture was treated with DNase I (Ambion) and precipitated with 1.25 M ammonium acetate/isopropanol. The precipitated RNA was applied to a NAP-5 column (GE Healthcare) to remove unincorporated NTPs, precipitated with 0.136 M sodium acetate/isopropanol, pelleted by centrifugation, washed with 70% ethanol, and used for the selection step. In each round of selection, RNA was heated to 85°C in binding buffer (10 mM Tris−HCl (pH7.6), 100 mM KCl) for 10 min and cooled in ice for 10 min. MgCl_2_ was then added to a concentration of 10 mM, and the RNA was incubated on BHQ1-immobilized affinity resin for 15 min at 4°C. The unbound RNA was collected and 10 μg of monomeric β-actin was added. The resulting solution was mixed with BHQ1-immobilized affinity resin and incubated for 30 min at 4°C. The resin was then drained and washed eight times with binding buffer to remove the non-binding species. Bound RNA species were eluted using binding buffer containing 2 μg of eluting antibody (BA3R/AC40), three times for 5 min each. The eluted fractions were pooled and precipitated with 0.136 M sodium acetate/isopropanol. Selected RNAs were reverse-transcribed using Prime Script reverse transcriptase (TAKARA), and the resulting cDNA was PCR-amplified with the forward and the reverse primers described above. The DNA templates were transcribed *in vitro*, and the resulting RNAs were subjected to the next round of selection. After 16 rounds of selection, enriched RNAs were reverse-transcribed and converted to dsDNA by PCR. The resulting DNAs were ligated into pUC19 at the EcoR I and BamH I sites and transformed to *Escherichia coli* strain DH5α. Several clones were isolated and sent for sequencing (Eurofins Genomics).

### Preparation of RNA for *in vitro* experiments

The dsDNAs for T7 transcription of RNA aptamers were PCR amplified using the recombinant pUC19 plasmid as a template, with T7 primer [5′-GCT AAT ACG ACT CAC TAT AGG GAT CCA AGC TTG T-3′], and reverse primer [5′-GAA TTC TCC GTC GAA AG-3′]. RNAs were transcribed from the dsDNA templates using T7 RNA polymerase from T7 MegaScript Kit (Ambion^®^) and purified using a NAP-5 column to remove unincorporated NTPs as described above.

### *In vitro* fluorescence measurement of RNA-protein interaction

RNA aptamer (3 μM) was denatured at 85°C for 10 min and cooled on ice for 10 min. The RNA was then mixed with β-actin (3 μM) in buffer (50 μl, 10 mM Tris−HCl (pH 7.6), 100 mM KCl) for 30 min at 25°C. The resulting RNA solution was mixed with a solution of conjugate **1** (final concentration: 1 μM) in a buffer (50 μl, 20 mM Tris−HCl (pH 7.6), 100 mM KCl, 10 mM MgCl_2_) and incubated at room temperature for 10 min. Fluorescence spectra of the sample were measured at the excitation wavelength of 620 nm and emission range of 640–800 nm. Competition experiments were performed by adding competitors together with β-actin. Antibodies were added at a final concentration of 3 μM, and peptides were added from 0 to 20 μM at 1% DMSO.

### Actin polymerization assay

Monitoring of actin polymerization was adapted according to the protocol described in the Actin Polymerization Biochem Kit (Cytoskeleton, Inc.), with some modifications. Briefly, 600 μl of G actin stock solution was prepared by dissolving 5 μM of actin (3% pyrene labeled) in G-buffer containing 2 mM Tris−HCl (pH 8.0), 0.1 mM CaCl_2_, and 0.1 mM ATP. The G actin stock solution was centrifuged at 21,500 × g at 4°C for 30 min. The supernatant was obtained, and 80 μl was taken and adjusted to binding buffer conditions (10 mM Tris–HCl (pH 7.6), 100 mM KCl and 10 mM MgCl_2_). RNA aptamer and conjugate **1** were added to a final concentration of 3 and 2 μM, respectively. The probe fluorescence of this solution was measured and taken as *t* = 0 min. ME buffer (final concentration 0.1 mM MgCl_2_ and 1 mM EGTA) was then added to the remaining G actin stock and incubated at room temperature for 2 min. KMEI was immediately added containing 50 mM KCl, 1 mM MgCl_2_, 1 mM EGTA and 10 mM imidazole (pH 7.0). A 100-μl aliquot was taken, and the fluorescence was immediately measured at the excitation wavelength of 365 nm and emission wavelength of 410 nm every 20 s for 15 min. An aliquot of the remaining, polymerizing G actin stock was taken every 3 min and mixed with the RNA aptamer and conjugate **1**. The solutions were adjusted to binding buffer conditions and the probe fluorescence was measured at the excitation wavelength of 620 nm and emission range of 640–800 nm.

### Peptide synthesis

The peptide epitope with sequence DDDIAALVVDNGSG (BA3R Antibody epitope) was prepared according to standard Fmoc solid phase peptide synthesis. Fmoc-Gly-OH was loaded on to 300 mg of Wang resin (1.03 mmol/g) using 2 eq. of DIC, 2 eq. of HOBt, and 0.1 eq. of DMAP in DCM/DMF for 2 h. Unreacted sites were capped with 0.5 eq. each of acetic anhydride and DIEA in DMF. Fmoc was removed by adding 20% piperidine in DMF in two 2 ml portions for 15 min each. Subsequent amino acids were loaded on the resin using 2 eq. each of HBTU and HOBt with 4 eq. of DIEA and mixed for 1.5 h. Success of couplings and deprotections was monitored by Kaiser test. Successful couplings were followed by capping and deprotection steps. This cycle was repeated until the whole sequence peptide was synthesized on the resin. The peptide was cleaved from the resin with a TFA cleavage cocktail (95% TFA/2.5% TIS/2.5% H_2_O) and precipitated with ice-cold ether. The crude product was then dried and subjected to HPLC purification and LC−MS analysis.

### Cell culture and DNA transfection

HeLa cells were maintained in Dulbecco's modified Eagle medium (Gibco^®^), supplemented with 1% penicillin, 100 μg/ml streptomycin sulfate, and 10% (v/v) fetal bovine serum (Biowest), at 37°C in a humidified incubator kept at 5% CO_2_. The cells were co-transfected with the RNA-aptamer expression plasmids and pSuper.gfp using FuGENE^®^ HD transfection reagent (Promega), according to the manufacturer's protocol. The RNA−aptamer sequences were cloned into pSuper vector (Oligoengine). Non-recombinant pSuper vector was used as a control.

### Fixed-cell Imaging of β-actin

HeLa cells were seeded on poly-d-lysine coated glass-bottom ViewPlate^®^-96F microplate (PerkinElmer, Inc.) at 7.5 × 10^3^ cells per well. After 24 h, cells were fixed with 4% paraformaldehyde solution (Muto Pure Chemicals Co., Ltd) at room temperature for 10 min, then washed twice with PBS and permeabilized with 0.5% Triton-X 100 for 10 min. The cells were then incubated with a blocking solution containing 10% dextran sulfate, 2 mM vanadyl-ribonucleoside complex, 0.01% salmon DNA, 30 μg yeast tRNA, 10 mM Tris−HCl (pH 7.6), and 100 mM KCl at room temperature for 1 h. The wells were washed twice with PBS and stained with 50 μl of 0.3 μM DNase I at room temperature for 30 min. Excess solution was removed, and the wells were washed twice with PBS. The cells were then incubated with 10 μM of P-TRap in binding buffer (10 mM Tris−HCl (pH7.6), 100 mM KCl and 10 mM MgCl_2_) at 4°C for 12 h, and then the solution was washed out twice with the binding buffer. The cells were stained with DAPI (10 μg/ml) in a binding buffer (50 μl) at room temperature for 20 min and treated with 5-μM conjugate **1** in the binding buffer (30 μl) at room temperature for 5 min. Excess conjugate **1** was washed out with the binding buffer, and the cells were observed on CV1000. Images were collected as 2 μm of vertical *z*-stack range (∼5 focal planes) and presented as MIP.

### Live-cell imaging of G-actin

On day 0, HeLa cells were seeded on a 35 mm 4-well Advanced TC glass bottom cell culture dish (Greiner Bio-One) at 5.0 × 10^5^ cells per well. On day 1, the cells were co-transfected with pSuper vector encoding each AcP-TRap1Ex and pSuper.gfp. On day 2, they were stained with Hoechst 33342 (1 mg/ml, Dojindo) in complete growth medium (500 μl) and incubated at 37°C for 20 min in a humidified 5% CO_2_ incubator. Conjugate **1** (final concentration: 5 μM) in a complete growth medium (100 μl) was then added and incubated at 37°C for 5 min in a humidified 5% CO_2_ incubator. Afterwards, the solution was replaced with fresh media, and the cells were observed on CV1000. Live-cell images were collected as 3.3 μm of vertical *z*-stack range (∼7 focal planes) and presented as MIP. Time-lapse images were taken every 5 min up to 1 h. For the observation of β-actin drug effects on fluorescent response, the cells were transfected as described above. The cells were stained with Hoechst 33342 and conjugate **2** and then treated with the actin drug (40 μM for cytochalasin B, 1 μM for latrunculin B, and 0.1 μM jasplakinolide at 0.5% DMSO). The live-cell images were collected immediately as 3.3 μm of vertical *z*-stack range (∼7 focal planes) and presented as MIP. The time-lapse images were taken as follows: Cytochalasin B 10-min intervals for up to 2 h; Latrunculin B 5-min intervals for up to 1 h; Jasplakinolide 3-min intervals for up to 30 min. The cells transfected with the non-recombinant pSuper were always prepared as control in every imaging experiment.

## RESULTS

### *In vitro* selection of a bifunctional RNA aptamer

**Figure 1.**
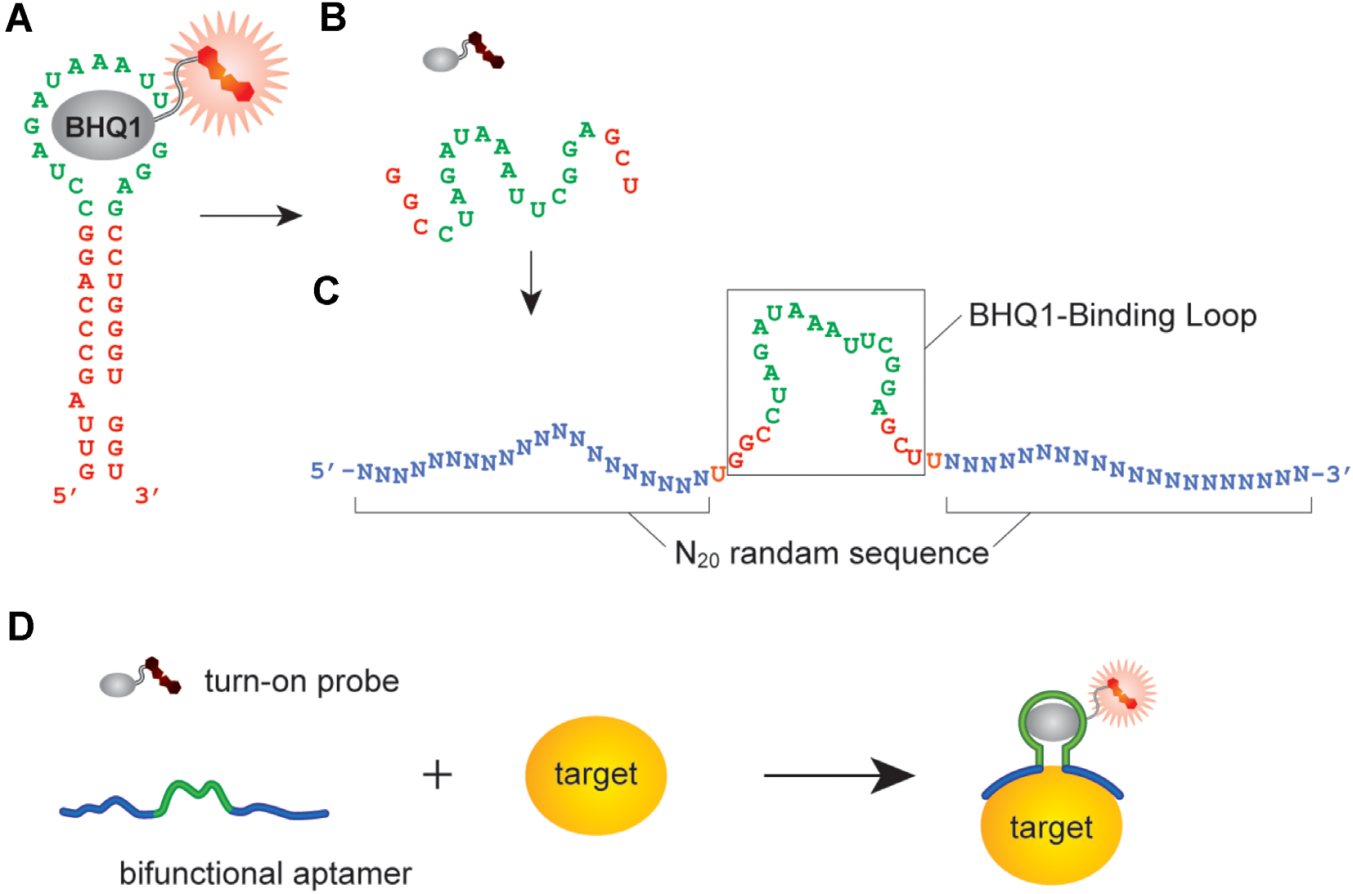
Design of a BHQ1-binding RNA aptamer responsive to a specific protein surface. (**A**) Secondary structure of the black-hole-quencher-1-binding aptamer (BHQ1 aptamer) with a ‘turn-on’ fluorescent probe. The BHQ1 aptamer comprises the stem structure (red) and the BHQ1-recognition loop (green). The BHQ1 aptamer restores the fluorescence intensity of the fluorescent probe by binding to the BHQ1 moiety of the probe. (**B**) Deletion of stem structure from the BHQ1 aptamer makes the BHQ1-recognition loop flexible, and the resulting short BHQ1-recognition loop alone cannot bind to the fluorescent probe. (**C**) Replacement of the stem structure in the BHQ1 aptamer with two 20-nt random sequences (blue) for *in vitro* selection of protein-targeting bifunctional aptamers. (**D**) A bifunctional aptamer bound to a target protein forms a short stem and the stable BHQ1-recognition loop structures and can bind to and restore the fluorescence of the probe.

To fluorescently label an endogenous protein in living cells, we designed a bifunctional RNA aptamer, whose functionality includes selective protein recognition and fluorescent probe binding. The probe binding is induced only upon recognizing its cognitive protein. If the probe is designed as a ‘turn-on’ fluorescent probe, the fluorescence of the probe is restored only on its cognitive protein, resulting in specific fluorescent labeling of the target protein (Figure 1). We used a black-hole-quencher-1-binding (BHQ1-binding) aptamer (33) (the *K*_d_ value of BHQ1-binding aptamer with conjugate **1** is 4.7 μM), which was previously discovered through *in vitro* selection (33–41). BHQ1-binding aptamer forms a simple stem–loop structure essential for BHQ1 recognition. The aptamer can bind to a fluorophore-BHQ1 turn-on probe (Figure 1A). Deletion of its stem region destabilizes the BHQ1-recognition loop, leading to a loss of BHQ1-binding activity (42) (Figure 1B). This highly sensitive structural property allowed us to design an RNA library for *in vitro* selection to obtain a bifunctional aptamer responsive to a specific native protein. Replacement of the stem structure in the BHQ1-binding stem–loop structure with two 20 random sequences would make the BHQ1-recognition loop flexible, and the resulting RNAs in the library alone cannot bind to BHQ1 (Figure 1C). The bifunctional aptamer, which restores BHQ1-binding activity exclusively on its target protein, can be isolated by efficient selection from the RNA library (Supplementary Figure S1).

**Figure 2.**
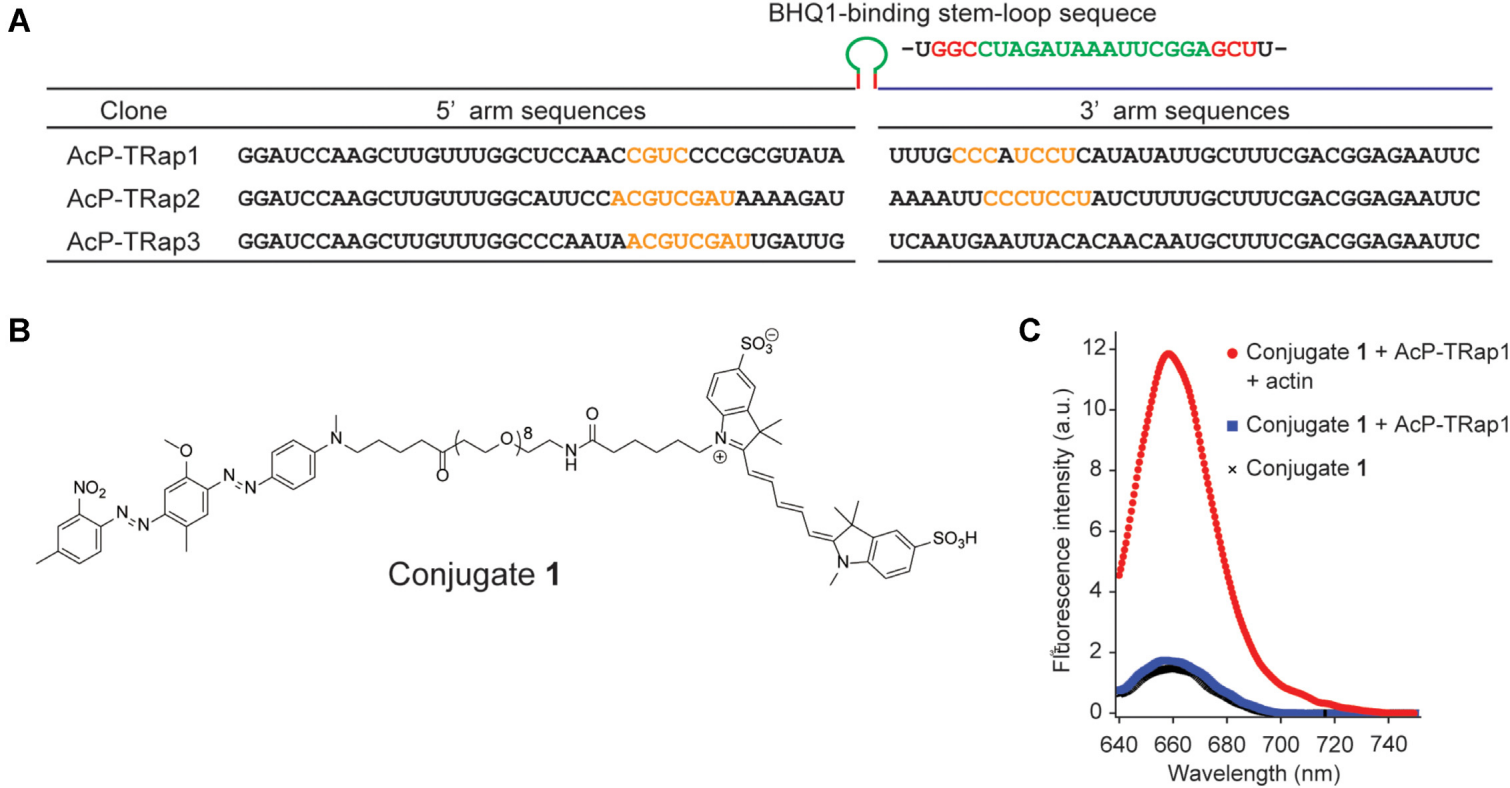
*In vitro* fluorescent labeling of β-actin. (**A**) Nucleotide sequences of actin-protein-targeting RNA aptamer (AcP-TRap). The conserved sequences of the AcP-TRap are shown in yellow. (**B**) Chemical structure of conjugate **1**. (**C**) Fluorescence intensities of conjugate **1** (final concentration: 1 μM) with the AcP-TRap1 in the presence or absence of a 3-μM β-actin (red and blue, respectively), and without AcP-TRap1 (black).

Through the use of the RNA library, *in vitro* selection was performed to isolate aptamers capable of targeting a native protein. As a target protein, we chose human β-actin due to its cellular abundance and the variety of known imaging methods for its validation (43). In the selection process, primarily to remove those RNA species which interact with BHQ1 in the absence of β-actin, the RNA library was treated with BHQ1-immobilized resins as a negative selection. The unbound RNAs were further mixed with β-actin to make a complex of RNA with β-actin, and the mixture was incubated again with BHQ1-immobilized resins. After extensive washing, BHQ1-binding RNA-β-actin complexes were competitively eluted with a monoclonal anti-β-actin antibody (clone BA3R). The eluted RNAs were utilized for reverse transcription PCR and transcription to generate the next RNA library. 16 rounds of selection narrowed down to three RNA species, which had conserved sequences (Figure 2A and Supplementary Figure S19). Their binding to β-actin was evaluated using a sulfo-Cy5-BHQ1 probe (conjugate **1**) (34) (Figure 2B). The three RNA species restored the fluorescence of conjugate **1** only in the presence of β-actin, indicating that the RNAs have bifunctional activities essential for protein labeling (Figure 2C and Supplementary Figure S2). Of the identified sequences, actin-protein-targeting RNA aptamer 1 (AcP-TRap1) exhibited the best response to the target β-actin with an apparent dissociation constant (*K*_d_) of 1.5 μM (Supplementary Figure S3, *K*_d_ values of AcP-TRap2, AcP-TRap3 and AcP-TRap4 are shown in Supplementary Figure S4); therefore, AcP-TRap1 was selected for further investigation.

### Evaluation of binding mode of AcP-TRap1

The proposed mechanism of aptamer binding involves the binding of two protein-targeting RNA ‘arms’ to the target protein. We attempted to truncate the arm sequence on both sides to ascertain whether we could optimize a minimal sequence for protein targeting. All the truncated aptamers showed reduced fluorescent response, indicating that the whole sequence of the protein-targeting arms is required for optimal β-actin binding (Supplementary Figure S5).

To investigate the target-recognition mechanism of AcP-TRap1, we performed binding assays with a peptide corresponding to the epitope of anti-β-actin antibody (BA3R). Increasing concentrations of the peptide were mixed with AcP-TRap1 and conjugate **1**, but no significant increase in fluorescent response was observed even up to 20 μM of the peptide (Supplementary Figure S6). Additionally, when β-actin was added to the peptide–aptamer solution (20-μM peptide mix), the fluorescent response of the aptamer to β-actin was restored (Supplementary Figure S6). These results indicate that AcP-TRap1 does not recognize the primary structure of the short peptide. The recognition mechanism was also evaluated by competitive binding analysis with anti-β-actin antibody (BA3R) (Supplementary Figure S7). Anti-β-actin antibody (BA3R), which was used for *in vitro* selection, disturbed the interaction of AcP-TRap1 with β-actin. These results suggested that AcP-TRap1 should recognize the three-dimensional surface of β-actin protein.

### *In situ* endogenous protein labeling

**Figure 3.**
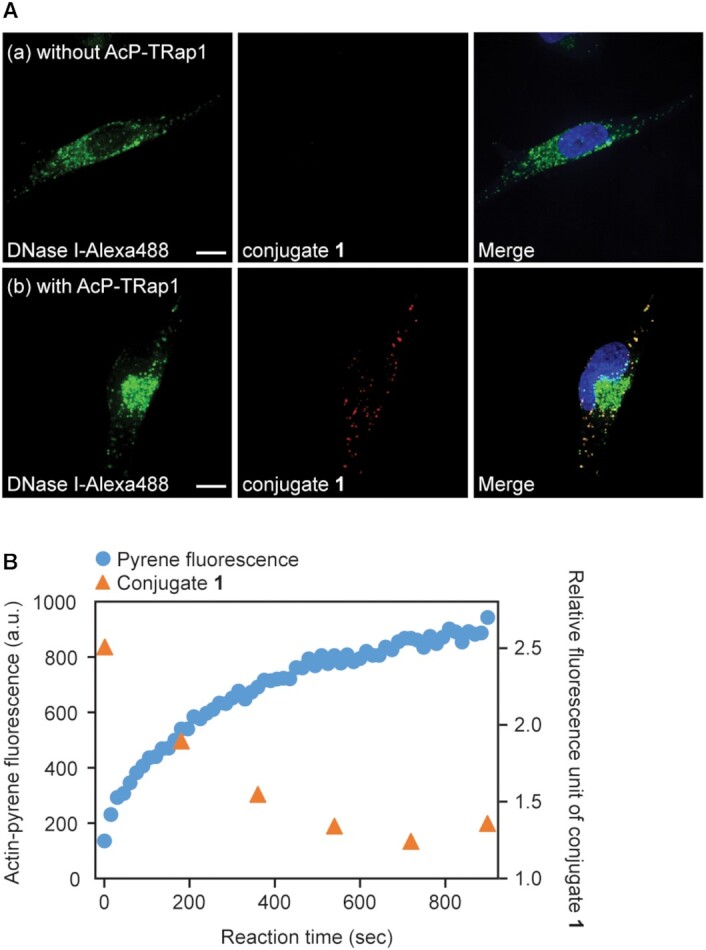
Actin staining with DNase I and AcP-TRap1. (**A**) Co-localization experiments between DNase-I-Alexafluor-488 (green) and AcP-TRap1-conjugate **1** (red). The nucleus was stained with Hoechst (blue in merge images). Scale bars are 10 μm. (**B**) *In vitro* actin polymerization assay. Formation of actin fibers was monitored by increasing pyrene fluorescence at 410 nm (blue dots). AcP-TRap1-conjugate **1** response (orange dots) was measured every 3 min.

To determine whether the aptamers are capable of fluorescent labeling of endogenous β-actin in the context of human cells, we proceeded to imaging experiments in fixed cells. To verify the specific labeling of actin protein, co-staining of actin was performed with AcP-TRap1 and phalloidin, a well-known actin stain in fixed cells. Interestingly, no overlap staining was observed between AcP-TRap1 and phalloidin (Supplementary Figure S8). Actin exists as both fibrous forms (F-actin) and globular forms (G-actin) in cells, and phalloidin specifically stains F-actin (44). Since AcP-TRap1 stains actin as strong fluorescent dots, it may be possible that AcP-TRap1 binds only to G-actin. To test this hypothesis, we performed co-staining experiments with DNase I, a protein known to bind selectively to G-actin in fixed cells (45). The fluorescent signals of aptamer overlapped with those of DNase I-Alexa488 probe, indicating that AcP-TRap1 recognizes preferentially to G-actin over F-actin (Figure 3A).

The recognition preference of AcP-TRap1 for G-actin was evaluated by *in vitro* actin polymerization assay. During actin polymerization reaction, the response of the aptamer was checked at different time points. As a result, the aptamer response showed an inverse relationship with the degree of actin polymerization (Figure 3B). This result clearly indicated that AcP-TRap1 shows preferential binding to G-actin over F-actin.

### Live-cell endogenous protein labeling

Based on the *in vitro* results, we applied the method to visualize endogenous actin in living HeLa cells. For live-cell imaging, AcP-TRap1 is expressed in cells with a short hairpin RNA expression vector that uses H1 RNA polymerase III promoter. Since AcP-TRap1 has a terminator sequence of RNA polymerase III, we optimized the AcP-TRap1 sequence suitable for expression in cells and examined its actin-targeting ability before the vector construction. The expression version of AcP-TRap1 (AcP-TRap1Ex) showed equivalent efficacy to the original AcP-TRap1 (Supplementary Figure S9). For live-cell actin imaging, the RNA expression vector encoding AcP-TRap1Ex was transfected with a GFP expression vector as a transfection control, and then the cells were treated with conjugate **2**, a cell-permeable derivative of conjugate **1** sixteen hours after transfection. The chemical structure and synthesis method of conjugate **2** is illustrated in Scheme S2. As we expected, strong fluorescence dots scattered throughout the cells expressing AcP-TRap1Ex was observed, whereas no significant background fluorescence was detected in the control cells (Supplementary Figure S10). Time-lapse images revealed that the fluorescent dots persisted for at least 1 h without a significant signal loss (Supplementary Videos S1 and S2).

**Figure 4.**
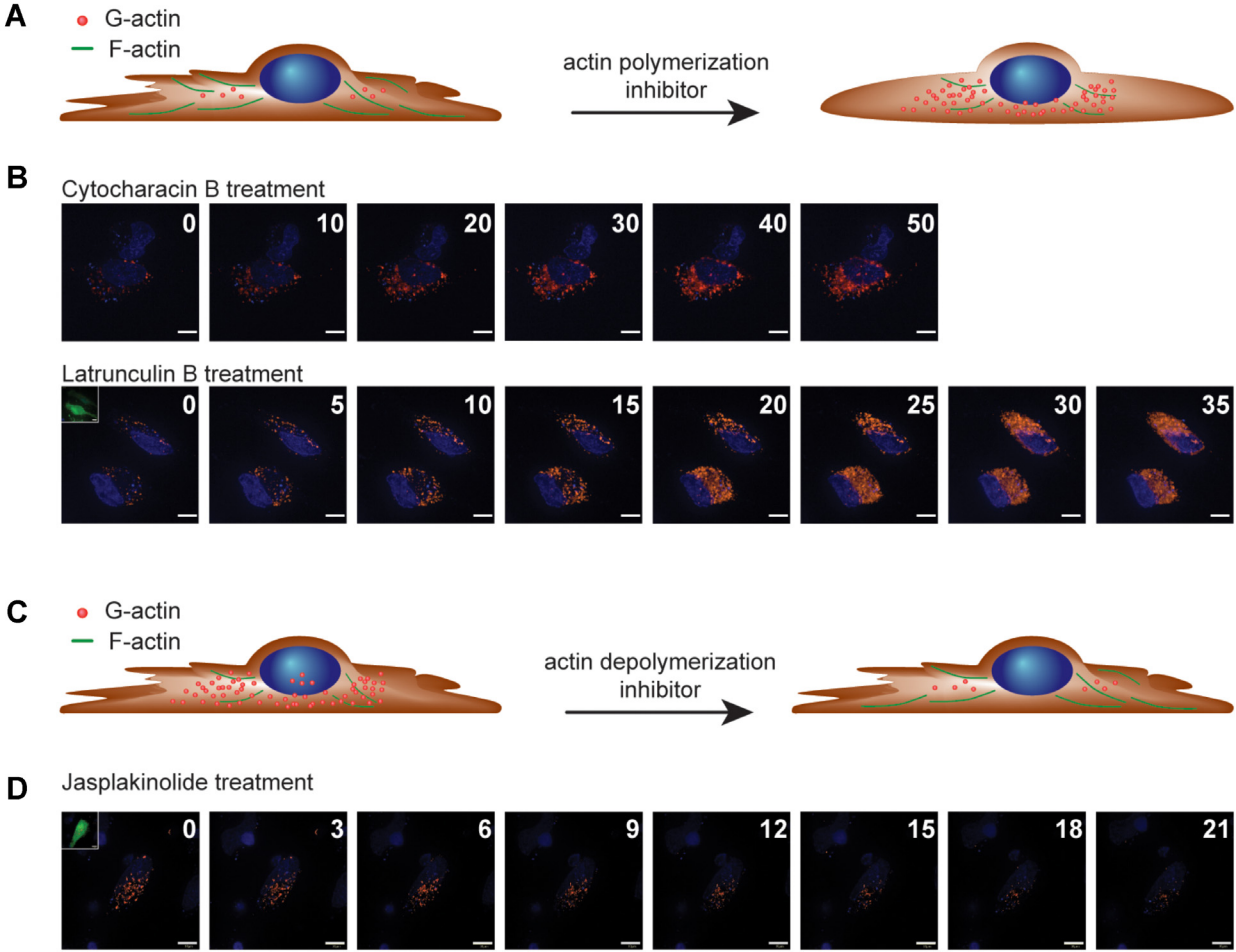
Time-dependent G-actin concentration changes in HeLa cells after treatment of actin drugs. Illustration of actin dynamics in cells after treatment of an actin polymerization inhibitor (**A**) or an actin depolymerization inhibitor (**C**), respectively. Time-lapse images of G-actin in live HeLa cells after cytochalasin B and latrunculin B treatment (**B**) and jasplakinolide treatment (**D**), respectively. All times are given in minutes. The nucleus was stained with Hoechst (blue). Scale bars, 10 μm.

This new protein-labeling method would allow us to visualize intracellular concentration change of endogenous proteins by time-lapse imaging. To investigate this hypothesis, we perturbed and observed G-actin dynamics inside cells with three known actin drugs: cytochalasin B (46, latrunculin B (47, and jasplakinolide (47). Precisely, cytochalasin B and latrunculin B are inhibitors of F-actin formation resulting in increasing the amount of G-actin in cells, while jasplakinolide is a stabilizer of F-actin, consequently lowering amounts of G-actin in cells (48). If AcP-TRap1Ex targets G-actin in cells, the number of detectable fluorescent dots should change by treating cells with the actin drugs. As expected, time-lapse images during the treatment with cytochalasin B and latrunculin B clearly showed a time-dependent increase in fluorescent response from AcP-TRap1Ex in cells (Figure 4A, B, Supplementary Figures S11–S12 and S14, and Supplementary Videos S3–S6). On the other hand, the fluorescent response reflects the expected decrease in G-actin concentration upon treatment with jasplakinolide, an F-actin stabilizer (Figure 4C and D, Supplementary Figures S13 and S14, Supplementary Videos S7 and S8). Overall, the results from actin-drug treatment all support that this protein-imaging method permits visualization of spatiotemporal endogenous protein dynamics in living cells.

### Surface-selective protein labeling

**Figure 5.**
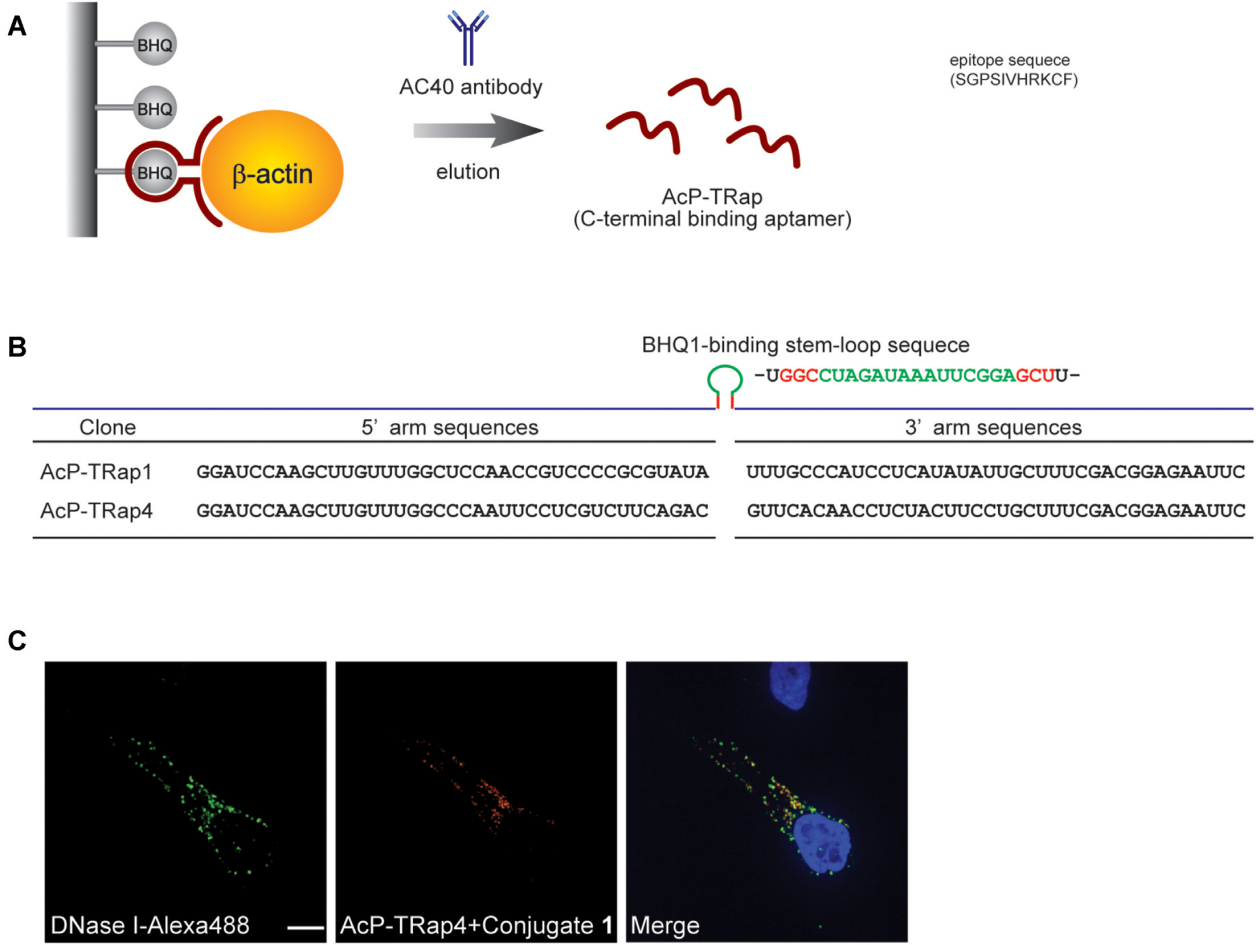
*In vitro* selection of AcP-TRap by using AC40 antibody. (**A**) Illustration of in vitro selection of AcP-TRap to β-actin with an anti-β-actin antibody (AC40). (**B**) Nucleotide sequences of AcP-TRap1 and AcP-TRap4. (**C**) Actin staining with DNase I and AcP-TRap4 in fixed HeLa cells. Co-localization experiments between DNase-I-Alexafluor-488 (green) and AcP-TRap4-conjugate **1** (red). The nucleus was stained with Hoechst (blue in merge images). Scale bar is 10 μm.

The method has a competitive edge in selecting the desired fluorescent labeling surface on a target protein through *in vitro* selection. To verify this advantage, we designed another cycle of *in vitro* selection for isolating a bifunctional aptamer that recognizes an orthogonal protein surface of β-actin. The *in vitro* selection was performed from the same initial RNA library as AcP-TRap1 but using a different eluting anti-β-actin antibody (clone AC40) that is specific for the C-terminal epitope of β-actin (Figure 5A). A bifunctional aptamer isolated from this *in vitro* selection should target β-actin at a unique binding surface. After 18 rounds of *in vitro* selection, we obtained a new aptamer (AcP-TRap4), which has different sequences from those of AcP-TRap1 in the β-actin targeting ‘arms’ (Figure 5B and Supplementary Figure S19). As expected, the AcP-TRap4 also showed β-actin targeting ability and competed for binding to β-actin with its corresponding eluting antibody (Supplementary Figure S15). Since AcP-TRap4 with conjugate **1** also stained its target proteins in fixed cells as strong fluorescent dots, we expected that the AcP-TRap4 would recognize preferentially to G-actin over F-actin. In fact, the fluorescent signals of aptamer overlapped with those of DNase I-Alexa488 probe (Figure 5C).

**Figure 6.**
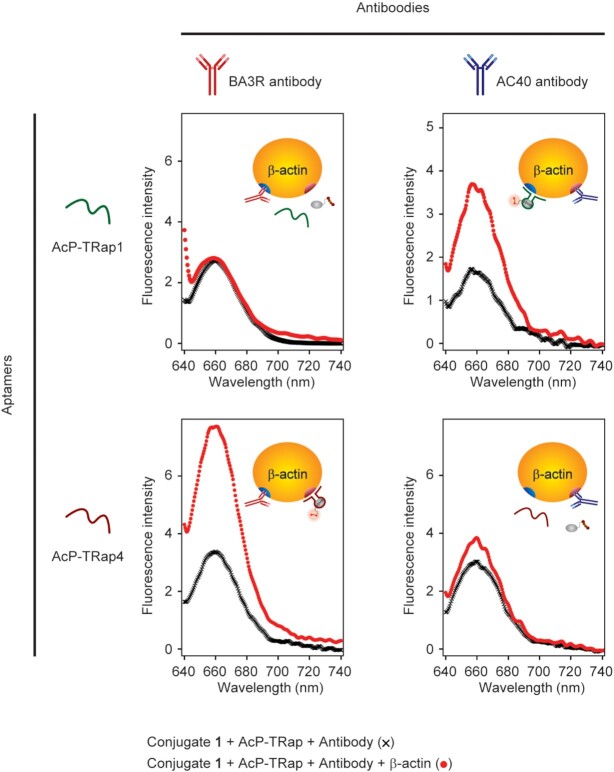
Evaluation of cross-reactivity of AcP-TRap1 and AcP-TRap4 against β-actin. Illustrations of inset show the putative complexes among AcP-TRap, β-actin, antibody, and conjugate **1**. Fluorescence intensities of conjugate **1** (final concentration: 1 μM) with antibodies, and the AcP-TRap1 or AcP-TRap4 (3 μM) in the absence or presence of β-actin (black and red, respectively).

To confirm the site specificity for β-actin recognition with each aptamer, we examined the cross-reactivity of each aptamer with each eluting antibody, respectively. Although both aptamers competed with their eluting antibodies for binding to β-actin, no detectable competition with opposite combinations (between AcP-TRap1 and antibody (AC40) or between AcP-TRap4 and antibody (BAR3)) was observed (Figure 6). The antibody competition analyses clearly elucidated that each aptamer can specifically recognize and label unique protein surfaces of β-actin. This lack of competition of cross-antibody-aptamers provides a possibility of obtaining an aptamer that can specifically label a target protein without disturbing its function. This protein-imaging technology is applicable to other endogenous proteins. We next attempted to identify a bifunctional aptamer that can label other endogenous proteins, a proto-oncogene KRAS (49,50). The *in vitro* selection for isolation of KRAS-protein-targeting RNA aptamers (KrP-TRap) was also performed from the same initial RNA library as β-actin targeting aptamers. A bifunctional aptamer, KrP-TRap1 obtained from the *in vitro* selection for KRAS restored the fluorescence of BHQ1-probe only in the presence of KRAS in *in vitro* binding assay (Supplementary Figure S16). Thus, these results also suggest that our protein labeling technology would be a promising tool for selective labeling of endogenous proteins in living cells.

## DISCUSSION

This non-covalent and cooperative labeling technology permits live-cell imaging of protein dynamics in an extremely selective manner. A bifunctional aptamer for a given target protein can easily be isolated with a well-designed conventional selection. We have demonstrated the effectiveness of this new protein-labeling method through imaging of β-actin in human cells. In live-cell imaging of β-actin, the method allowed us to monitor the concentration change of G-actin through response to known chemical stimuli. This is the first reported imaging tool specific for G-actin in mammalian living cells. The identity of G-actin was confirmed independently by co-staining with fluorophore-conjugated DNase I in fixed cells. Although the fluorescent signals well overlapped with those of the fluorescent DNase I, the number of fluorescent spots observed was less than that of the fluorescent DNase I. One possible reason behind this is that AcP-TRap may not recognize G-actin that exists as a complex with other partner proteins. To verify this hypothesis, we examined the binding competition of AcP-TRap1 with three well-known G-actin-binding proteins, cofilin (51, profilin (52, and DNase I (53). Both cofilin and profilin disturbed the interaction of AcP-TRap1 with β-actin, although the AcP-TRap1 can still bind to β-actin even in the presence of DNase I (Supplementary Figure S17). These results suggest the possibility that AcP-TRap would not be able to label cofilin- or profilin-bound G-actin in cells. Since G-actin is known to be complexed with over 25 other proteins, including cofilin and profilin in mammalian cells (54, G-actin might not solely exist as monomers in cells, which consequently leads to the labeling of only a subset of G-actin by our method. If *in vitro* selection is performed for such a complex, a highly specific P-TRap for the complex of G-actin and the partner protein could be isolated. These results reinforce the fact that this new method excels at protein labeling in an exquisitely selective manner. However, the binding of AcP-TRap to G-actin may affect the G-actin localization or the F-actin formation. Further validation of the new method might permit the detection of a variety of proteins without affecting their dynamics.

We have developed a live-cell protein imaging technology that uses a protein-targeting RNA aptamer with a turn-on fluorescent probe. One notable advantage of the technology is that it simply requires practices of commonly used cell biology techniques. A highly selective P-TRap for a specific target protein can be easily isolated by well-designed *in vitro* selection. A cell-permeable turn-on probe can simply be added to culture media to visualize protein dynamics. As for the modularity of the method, the probe-binding stem-loop structure can be replaced by other probe-binding structures (55–58) (Supplementary Figure S18). These advantages combined together allow for the design of bifunctional aptamers capable of binding to various probes for the simultaneous visualization of molecular dynamics of multiple endogenous proteins in a single cell. Overall, the present study successfully demonstrates a bifunctional-aptamer-based method that permits spatiotemporal imaging of specific endogenous proteins in living mammalian cells.

## Supplementary Material

gkab839_Supplemental_Files

## Notes

Present address: Kenji Yatsuzuka, Faculty of Pharmaceutical Sciences, Doshisha Women's College of Liberal Arts, Kyotanabe, Kyoto 610-0395, Japan.
