# Supplementary material for: RNA-based cooperative protein labeling that permits direct monitoring of the intracellular concentration change of an endogenous protein
Source: Nucleic Acids Res. 2021 Sep 28;49(22):e132. doi: 10.1093/nar/gkab839 (PMC8682759; doi:10.1093/nar/gkab839)
Supplement: gkab839_Supplemental_Files [file gkab839_supplemental_files.zip › SI.pdf]

SUPPLEMENTARY INFORMATION

RNA-based cooperative protein labeling that permits direct monitoring of the intracellular concentration change of an endogenous protein

Kathleen Beverly Alog Pe,[1] Kenji Yatsuzuka,[1] Hayase Hakariya,[1] Tomoki Kida,[3] Yousuke Katsuda,[2,3] Masatora Fukuda[4] and Shin-ichi Sato[1,]*

[1]Institute for Chemical Research and [2]Institute for Integrated Cell-Material Sciences (WPI-iCeMS), Kyoto University, Uji, Kyoto 611-0011, Japan.
[3]Division of Materials Science and Chemistry, Faculty of Advanced Science and Technology, Kumamoto University, 2-39-1 Kurokami, Chuo-ku, Kumamoto 860-8555, Japan
[4]Department of Chemistry, Faculty of Science, Fukuoka University, 8-19-1 Nanakuma, Jonan-ku, Fukuoka, Fukuoka
814-0180, Japan.

***E-mail: ssato@scl.kyoto-u.ac.jp (S.S.)

Supplementary Contents

Supplementary Methods 3

Supplementary Note 1. 3

Supplementary Note 2. 3

Supplementary Note 3. 4

Supplementary Schemes 5

Scheme S1 5

Scheme S2 6

Scheme S3 6

Scheme S4 7

Supplementary Figures 8

Supplementary Figure S1. 8

Supplementary Figure S2. 9

Supplementary Figure S3. 10

Supplementary Figure S4. 11

Supplementary Figure S5. 12

Supplementary Figure S6. 13

Supplementary Figure S7. 14

Supplementary Figure S8. 15

Supplementary Figure S9. 16

Supplementary Figure S10. 17

Supplementary Figure S11. 18

Supplementary Figure S12. 19

Supplementary Figure S13. 20

Supplementary Figure S14. 21

Supplementary Figure S15. 22

Supplementary Figure S16. 23

Supplementary Figure S17. 24

Supplementary Figure S18. 25

Supplementary Figure S19. 26

Supplementary Videos 27

Supplementary References 28

Supplementary Notes

Supplementary Note 1. Reagents

Buffers and salt solutions for *in vitro* experiments (Tris-HCl buffers, NaCl, KCl, and $MgCl_2$) were purchased from Nacalai Tesque Inc. (Kyoto, Japan). Antibodies against β-actin (clone numbers BA3R and AC40) were purchased from Abcam (Cambridge, United Kingdom). Primers and oligonucleotides were ordered from Invitrogen (California, USA). β-actin and Alexafluor488-labelled phalloidin were ordered from Cytoskeleton Inc. (Colorado, USA). Alexa-fluor labelled DNase 1 was ordered from Thermo Fisher Scientific (Massachusetts, USA). PBS and media for cell experiments were purchased from Gibco (Massachusetts, USA). Wang resin and amino acids for solid phase peptide synthesis were purchased from Watanabe Chemical Industries (Hiroshima, Japan). Other chemical reagents for peptide synthesis were purchased from Wako Pure Chemicals (Tokyo, Japan).

Supplementary Note 2. Chemical synthesis of conjugates 1 and 2.

General. The solvents and chemicals for chemical syntheses were used as purchased, with no further purification. Fmoc-amido-dPEG$_4$™-acid was purchased from Quanta BioDesign, Ltd. (Ohio, USA). Cy5-NHS-ester, sulfo-Cy5-NHS-ester and sulfo-Cy3-NHS-ester were purchased from Lumiprobe Co. (Florida, USA). BHQ-1 Amine was purchased from Biosearch Technologies, Inc. (California, USA). TAMRA-SE was purchased from AAT Bioquest, Inc. (California, USA). All other solvents and chemicals were purchased from Tokyo Chemical Industry Co., Ltd. (Tokyo, Japan) or Nacalai Tesque, Inc. (Kyoto, Japan). High-performance liquid chromatography (HPLC) was performed with a combined system of Shimadzu CTO-10AC, LC-10AD (pump x2), CBM-20A (controller), and SPD-M20A (detector). The synthetic molecules were purified by reversed-phase HPLC. HPLC conditions were as follows: GL science Inertsil® ODS-3 column (5 μm, 20 x 100 mm); solvent gradient, A, 0.1% trifluoroacetic acid (TFA) in H_2O; B, 0.1% TFA in acetonitrile with gradient indicated below; flow rate, 4.0 mL/min; detector, 254/280/530/560 nm. HPLC grade reagents were used. Low-resolution mass spectra were obtained using a Shimadzu LCMS-2010 in ESI mode. High-resolution mass spectra were obtained using a JEOL MStation JMS-700V in FAB mode.

Supplementary Note 3. Peptide synthesis

The peptide epitope with sequence DDDIAALVVDNGSG (BA3R antibody epitope) was prepared according to standard Fmoc solid phase peptide synthesis. Fmoc-Gly-OH was loaded on to 300 mg of Wang resin (1.03 mmol/g) using 2 eq. of DIC, 2 eq. of HOBt and 0.1 eq. of DMAP in DCM/DMF for 2 hours. Unreacted sites were capped with 0.5 eq. each of acetic

anhydride and DIEA in DMF. Fmoc was removed by adding 20% piperidine in DMF in two 2 mL portions for 15 minutes each. Subsequent amino acids were loaded on the resin using 2 eq. each of HBTU and HOBt with 4 eq. of DIEA and mixed for 1.5 hours. Success of couplings and deprotections were monitored by Kaiser tests. Successful couplings were followed by capping and deprotection steps. This cycle was repeated until the whole sequence peptide was synthesized on the resin. The peptide was cleaved from the resin with a TFA cleavage cocktail (95% TFA/2.5% TIS/ 2.5% H_2O) and precipitated with ice-cold ether. The crude product was then dried and subjected to HPLC purification and LC-MS analysis.

Scheme S1: Synthesis of conjugate 1

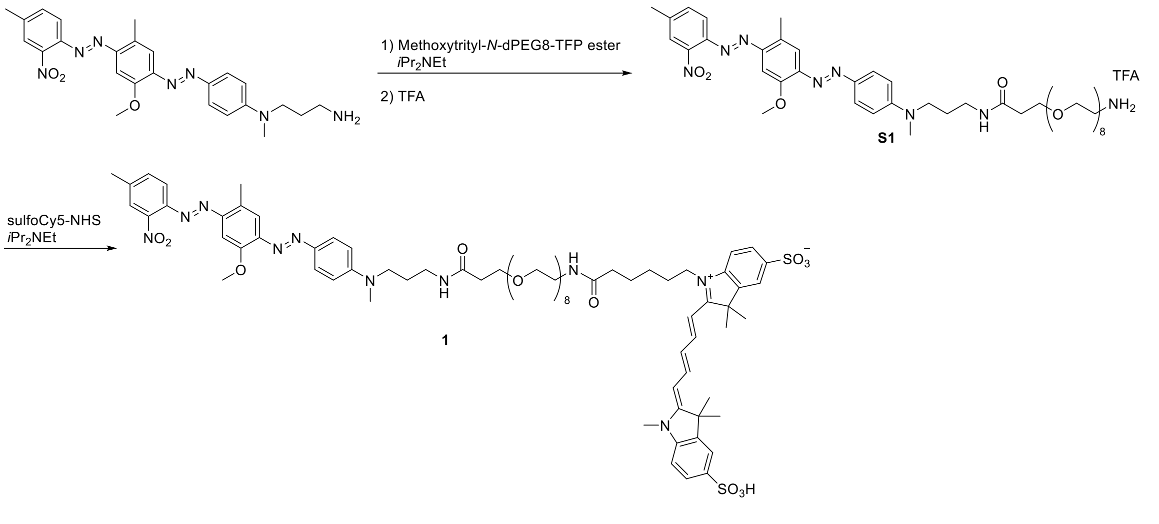

Methoxytrityl-*N*-dPEG®$_8$-TFP ester (12.9 mg, 15 μmol) was added to a solution of BHQ1 amine (4.75 mg, 10 μmol) and *i*Pr$_2$NEt (6.80 μL, 40 μmol) in DMSO (50 μL). After stirring for 3 h at room temperature, H_2O (300 μL) and hexane (500 μL) were added to the mixture, and the resulting mixture was centrifuged (21,500 ×g for 10 min). After removal of supernatant, residual oil was washed with H_2O (500 μL). The residue was dried *in vacuo*. The dried residue was dissolved in TFA (400 μL). After stirring for 2 h at room temperature, the mixture was dried *in vacuo*. Resulting crude mixture was purified by preparative HPLC (0–3 min: B, 55%; 3–23 min: B, 55–65% at RT, t_R = 20.6 min) gave intermediate **S1** (57% yield). ^{1}H-NMR (600 MHz, DMSO-*d*$_6$) δ: 7.95–7.93 (1H, m), 7.92 (1H, t, *J* = 6.0 Hz), 7.90 (2H, d, *J* = 9.6 Hz), 7.76 (1H, d, *J* = 8.4 Hz), 7.71 (3H, brs), 7.70–7.67 (1H, m), 7.51 (1H, d, *J* = 0.6 Hz), 7.29 (1H, s), 6.85 (2H, d, *J* = 9.6 Hz), 3.92 (3H, s), 3.62 (2H, t, *J* = 6.6 Hz), 3.59 (2H, t, *J* = 4.8 Hz), 3.57–3.46 (30H, m), 3.13 (2H, q, *J* = 6.6 Hz), 3.06 (3H, s), 2.97 (2H, q, *J* = 6.0 Hz), 2.63 (3H, d, *J* = 0.6 Hz), 2.54 (3H, s), 2.34 (2H, t, *J* = 6.6 Hz), 1.72 (2H, quint, *J* = 7.2 Hz). ^{13}C-NMR (150 MHz, DMSO-*d*$_6$) δ: 169.99, 154.25, 151.68, 150.02, 146.29, 144.62, 143.43, 142.52, 142.19, 133.81, 132.12, 125.39, 124.18, 119.98, 118.38, 111.35, 99.09, 69.65, 69.63, 69.61, 69.58, 69.55, 69.51, 69.43, 66.79, 66.56, 55.84, 49.30, 39.97, 38.20, 36.12, 36.11, 26.44, 20.58, 16.16. MS (ESI) calculated for $C_{44}H_{67}N_8O_{12}$ [M+H]$^+$: 899.4873 was 899.4873.

SulfoCy5-NHS (0.248 mg, 0.33 μmol) was added to a solution of (**S1** (0.25 μmol) and *i*Pr$_2$NEt (0.17 μL, 1.0 μmol) in DMSO (20 μL). After stirring for 12 h at room temperature, the mixture was diluted with acetonitrile/H_2O/TFA (600 μL, 47.5:47.5:5 solution) and the mixture was purified by preparative HPLC (0–3 min: B, 45%; 3–21 min: B, 45–61% at RT, t_R = 19.2 min) gave **1** (79% yield). ^{1}H-NMR (600 MHz, DMSO-*d*$_6$) δ: 8.34 (2H, dd, 13.2, 13.2), 7.94–7.90 (2H, m), 7.82–7.78 (1H, m), 7.81 (1H, s), 7.78 (2H, d, *J* = 9.0 Hz), 7.76 (1H, d, *J* = 8.4 Hz), 7.69–7.66 (1H, m), 7.66–7.62 (2H, m), 7.50 (1H, d, *J* = 0.6 Hz), 7.33–7.28 (2H, m), 7.28 (1H, s), 6.84 (2H, d, *J* = 9.0 Hz), 6.55 (1H, dd, *J* = 13.2, 13.2 Hz), 6.29 (1H, d, *J* = 13.2 Hz), 6.24 (1H, d, *J* =

13.2 Hz), 4.06 (2H, t, J = 7.2 Hz), 3.91 (3H, s), 3.61 (2H, t, J = 6.6 Hz), 3.58 (3H, s), 3.53–3.46 (32H, m), 3.40–3.30 (16H, m) 3.19–3.11 (4H, m), 3.05 (3H, s), 2.62 (3H, s), 2.33 (2H, t, J = 6.6 Hz), 2.05 (2H, t, J = 7.2 Hz), 1.75–1.65 (2H, m), 1.53 (2H, quint, J = 7.2 Hz), 1.37–1.29 (2H, m), 1.28–1.23 (2H, m). MS (ESI) calculated for $C_{76}H_{102}N_{10}NaO_{19}S_2$ $[M+Na]^{2+}$: 784.3274 was 784.3270.

Scheme S2: Synthesis of conjugate 2

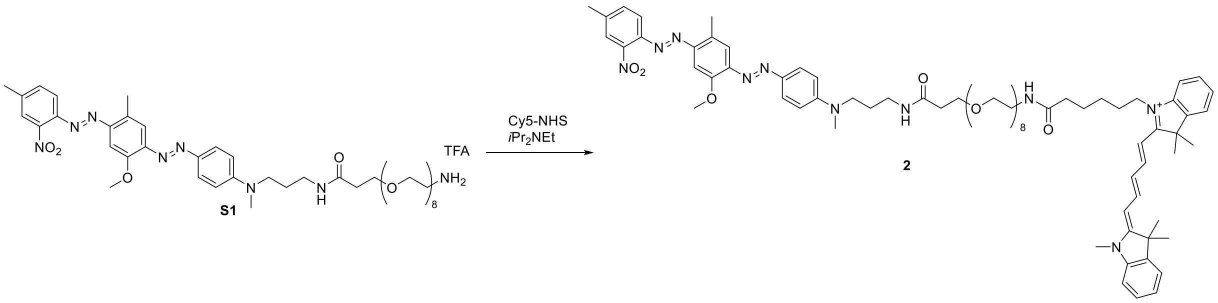

Cy5-NHS (0.370 mg, 0.60 µmol) was added to a solution of **S1** (0.50 µmol) and *i*Pr$_2$NEt (0.34 µL, 2.0 µmol) in DMSO (20 µL). After stirring for 12 h at room temperature, the mixture was diluted with acetonitrile/H_2O/TFA (600 µL, 47.5:47.5:5 solution) and the mixture was purified by preparative HPLC (0–3 min: B, 55%; 3–33 min: B, 55–80% at RT, t_R = 29.1 min) gave **2** (76% yield). MS (ESI) calculated for $C_{76}H_{103}N_{10}NaO_{13}$ $[M+Na]^{2+}$: 693.3796 was 693.3791.

Scheme S3: Synthesis of conjugate 3

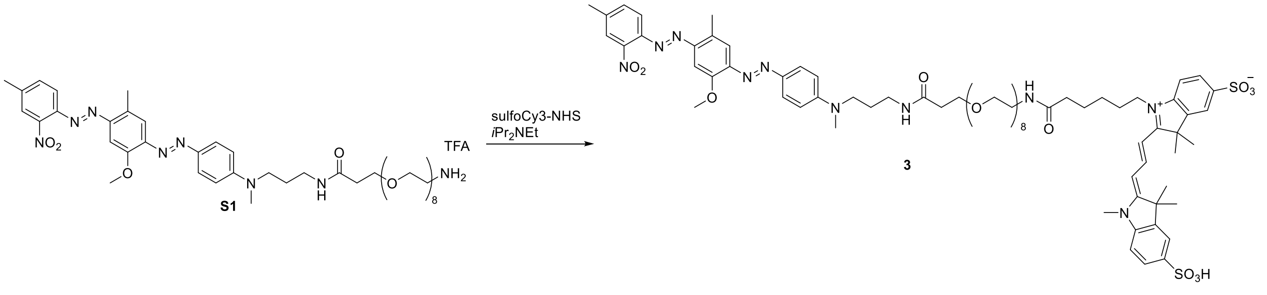

sulfoCy3-NHS (0.551 mg, 0.75 µmol) was added to a solution of **S1** (0.50 µmol) and *i*Pr$_2$NEt (0.34 µL, 2.0 µmol) in DMSO (20 µL). After stirring for 12 h at room temperature, the mixture was diluted with acetonitrile/H_2O/TFA (600 µL, 47.5:47.5:5 solution) and the mixture was purified by preparative HPLC (0–3 min: B, 45%; 3–20.5 min: B, 45–58% at RT, t_R = 22.0 min) gave **3** (22% yield). MS (ESI) calculated for $C_{74}H_{100}N_{10}Na_2O_{19}S_2$ $[M+2Na]^{2+}$: 771.3196 was 771.3183.

Scheme S4: Synthesis of conjugate 4

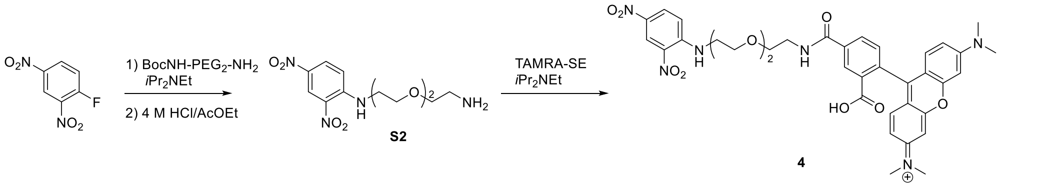

2,4-Dinitrofluorobenzen (106 μL 0.85 mmol) was added to a solution of BocNH-PEG$_2$-NH$_2$ (233 mg, 0.94 mmol) and *i*Pr$_2$NEt (288 μL 1.7 mmol) in CH_2Cl_2 (5 mL). After stirring for 2 h at room temperature, 0.2 M HCl aq. (20 mL) was added to the mixture, and the resulting mixture was extracted with CH_2Cl_2 (10 mL × 3). The combined organic layer was washed with brine (10 mL), dried over Na_2SO_4, filtered, and evaporated. The residue was purified by silica gel column chromatography using hexane/AcOEt (77:23 to 19:81) as eluent, to give DNB-peg$_2$-NHBoc (322mg, 92% yield). ^{1}H-NMR (300 MHz, $CDCl_3$) δ: 9.15 (1H, d, *J* = 2.8 Hz), 8.82 (1H, brs) 8.29 (1H, dd, *J* = 2.8, 9.3), 6.94 (1H, d, *J* = 9.3), 4.97 (1H, brs), 3.84 (2H, t, *J* = 5.4), 3.75–3.50 (m, 8H), 3.33 (2H, q, *J* = 4.8), 1.43 (9H, s).

DNB-peg$_2$-NHBoc (322 mg, 0.78 mmol) was dissolved in 4 M HCl/AcOEt (5 mL) and stirred for 3 h at room temperature. The resulting solid was collected by filtration to give **S2** as a HCl salt (236 mg, 86% yield). ^{1}H-NMR (300 MHz, DMSO-*d*$_6$) δ: 8.87 (1H, d, *J* = 2.4 Hz), 8.87–8.83 (1H, m), 8.28 (1H, dd, *J* = 2.4, 9.6 Hz), 7.97 (3H, brs), 7.30 (1H, d, *J* = 9.6 Hz), 3.75–3.66 (4H, m), 3.65–3.55 (6H, m), 2.94 (2H, q, *J* = 5.4 Hz). MS (ESI) calculated for $C_{12}H_{19}N_4O_6$ $[M+H]^+$: 315.1299 was 315.1299.

DNB-peg$_2$-NHBoc (322 mg, 0.78 mmol) was dissolved in 4 M HCl/AcOEt (5 mL) and stirred for 3 h at room temperature. The resulting solid was collected by filtration to give **S2** as a HCl salt (236 mg, 86% yield). ^{1}H-NMR (300 MHz, DMSO-*d*$_6$) δ: 8.87 (1H, d, *J* = 2.4 Hz), 8.87–8.83 (1H, m), 8.28 (1H, dd, *J* = 2.4, 9.6 Hz), 7.97 (3H, brs), 7.30 (1H, d, *J* = 9.6 Hz), 3.75–3.66 (4H, m), 3.65–3.55 (6H, m), 2.94 (2H, q, *J* = 5.4 Hz). MS (ESI) calculated for $C_{12}H_{19}N_4O_6$ $[M+H]^+$: 315.1299 was 315.1299.

TAMRA-SE (0.158 mg, 0.30 μmol) was added to a solution of **S2** (0.0875 mg, 0.25 μmol) and *i*Pr$_2$NEt (0.170 μL 1.0 μmol) in DMSO (20 μL). After stirring for 12 h at room temperature, the mixture was diluted with acetonitrile/H_2O/TFA (600 μL, 47.5:47.5:5 solution) and the mixture was purified by preparative HPLC (0–3 min: B, 40%; 3–21 min: B, 40–49% at RT, t_R = 20.9 min) gave **6** (84% yield). ^{1}H-NMR (600 MHz, DMSO-*d*$_6$) δ: 13.31 (1H, brs), 8.92–8.87 (1H, m), 8.85 (1H, t, *J* = 5.4 Hz), 8.83 (1H, d, *J* = 2.4 Hz), 8.63 (1H, brs), 8.26 (1H, d, *J* = 7.8 Hz), 8.23 (1H, ddd, *J* = 0.6, 2.4, 9.6 Hz), 7.51 (1H, brs), 7.26 (1H, d, *J* = 9.6 Hz), 7.23–6.64 (5H, m), 6.53 (1H, m), 3.72 (2H, t, *J* = 5.4 Hz), 3.65 (2H, t, *J* = 5.4 Hz), 3.65–3.59 (4H, m), 3.59 (2H, t, *J* = 6.0 Hz), 3.53–3.45 (2H, m), 3.22 (12H, brs). MS (ESI) calculated for $C_{37}H_{39}N_6O_{10}$ $[M]^+$: 727.2722 was 727.2714.

Supplementary Figures

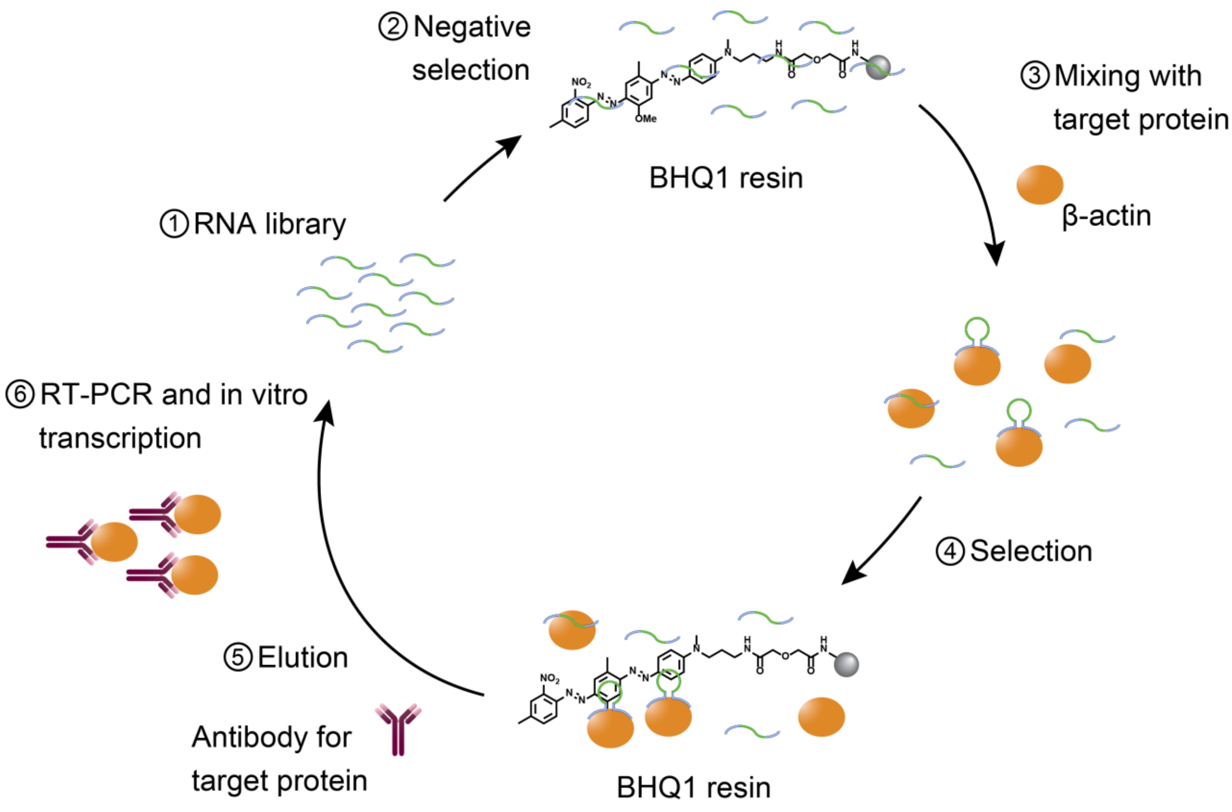

Supplementary Figure S1: *In vitro* selection of AcP-TRap.

Scheme of *in vitro* selection strategy for AcP-TRap. Negative selection step was incorporated into the selection protocol before selection step to remove RNA species which bind to BHQ1 without β-actin.

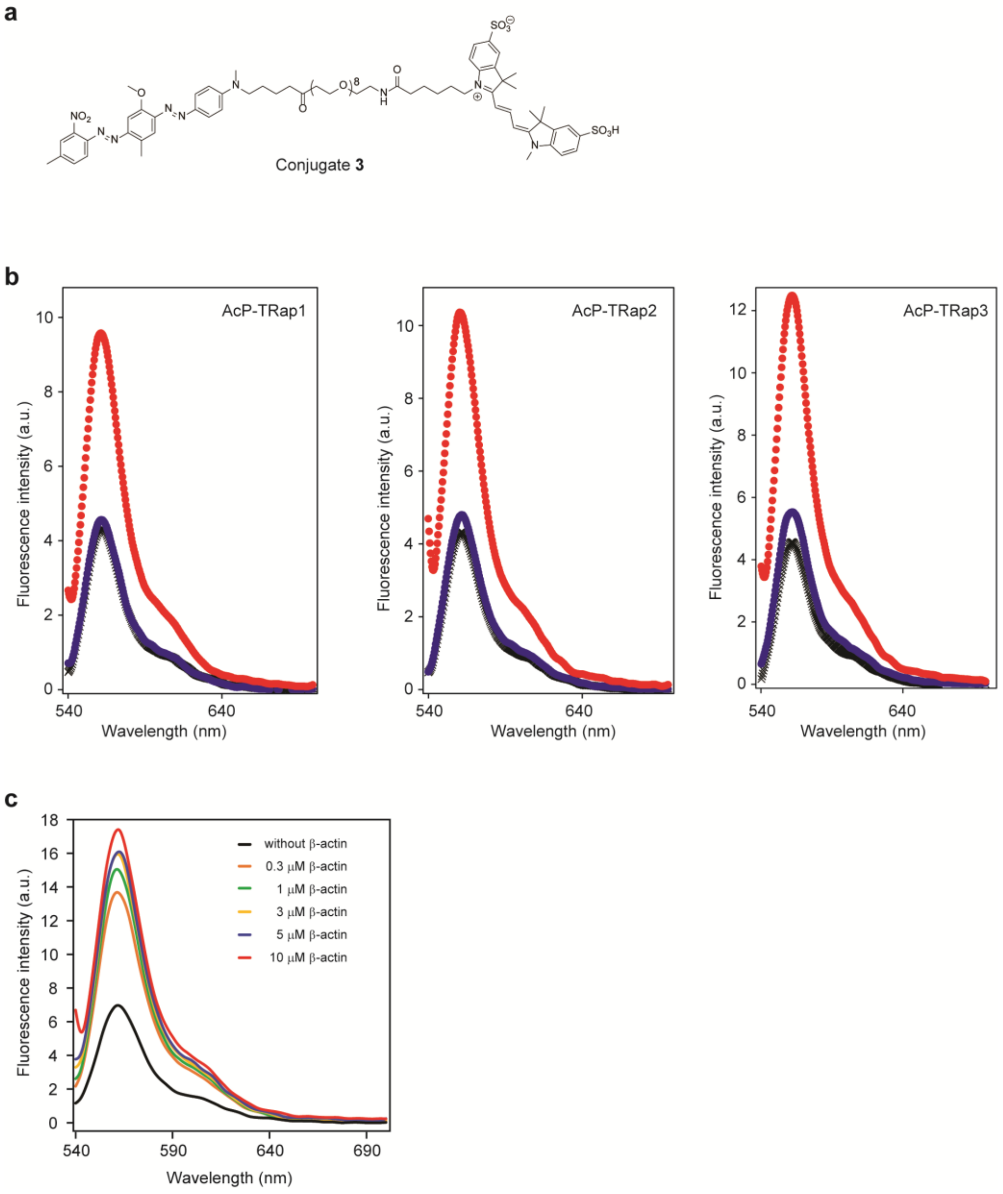

Supplementary Figure S2: Fluorescent protein labeling with AcP-TRaps.

(a) Chemical structure of conjugate **3**(supplementary ref.1). **(b)** Fluorescence intensities of conjugate **3** (final concentration: 1 μM) with the AcP-TRap1, AcP-TRap2 and AcP-TRap3 (3 μM) in the presence or absence of a 3-μM β-actin (blue and red, respectively), and without AcP-TRaps (black). The AcP-TRaps increase the fluorescence intensities of conjugate **3** upon binding with β-actin. **(c)** Fluorescence of the probe was restored by AcP-TRap1 with β-actin in a β-actin concentration-dependent manner.

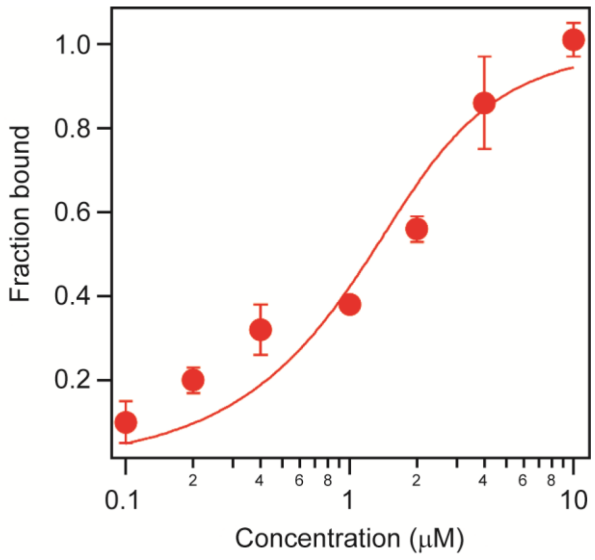

Supplementary Figure S3: K_d of AcP-TRap1 with β-actin.

Determination of K_d value in 10 mM Tris-HCl buffer (pH 7.6) containing 100 mM KCl and 10 mM $MgCl_2$. Semilogarithmic plots showing the fraction of AcP-TRap1 bound to β-actin at room temperature. The apparent K_d value of AcP-TRap with β-actin was calculated to be 1.5 μM (±0.4 μM).

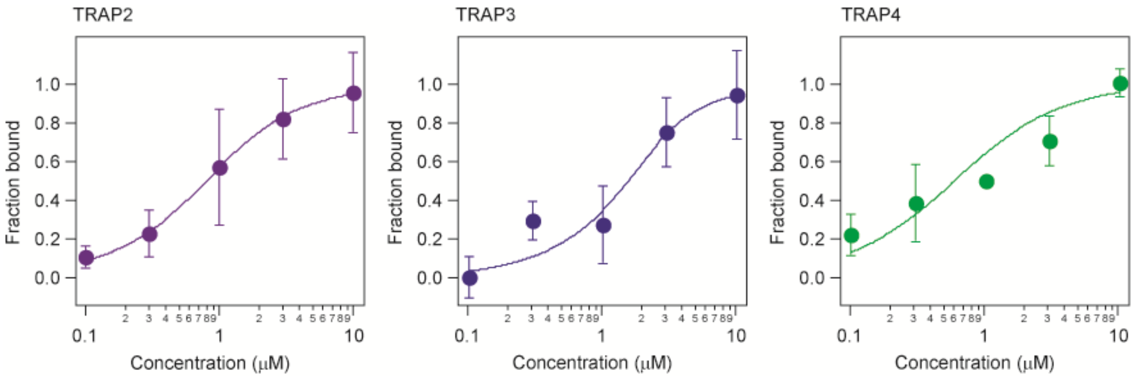

Supplementary Figure S4: K_d of AcP-TRap2, AcP-TRap3 and AcP-TRap4 with β-actin. Determination of K_d value in 10 mM Tris-HCl buffer (pH 7.6) containing 100 mM KCl and 10 mM $MgCl_2$. Semilogarithmic plots showing the fraction of AcP-TRaps bound to β-actin at room temperature. The apparent K_d values of AcP-TRap2, AcP-TRap3 and AcP-TRap4 with β-actin was calculated to be 0.6±0.1, 1.0±0.3 and 0.2±0.3 μM, respectively.

a

BHQ1-binding stem-loop sequece

-UGGCCUAGAUAAAUUCGGAGCUU-

Clone	5' arm sequences	3' arm sequences
AcP-TRap1	GGAUCCAAGCUUGUUUGGCUCCAACCGUCCCCGCGUAUA	UUUGCCCAUCCUCAUAUAUUGCUUUCGACGGAGAAUUC
truncate1	GUCCAACCGUCCCCGCGUAUA	UUUGCCCAUCCUCAUAUAUU
truncate2	GUUGUUUGGCUCCAACCGUCCCCGCGUAUA	UUUGCCCAUCCUCAUAUAUUGCUUUCGA
truncate3	GUUGUUUGGCUCCAACCGUCCCCGCGUAUA	UUUGCCCAUCCUCAUAUAUUGCUUUCGACGGAGAAUUC
truncate4	GGAUCCAAGCUUGUUUGGCUCCAACCGUCCCCGCGUAUA	UUUGCCCAUCCUCAUAUAUUGCUUUCGA

b

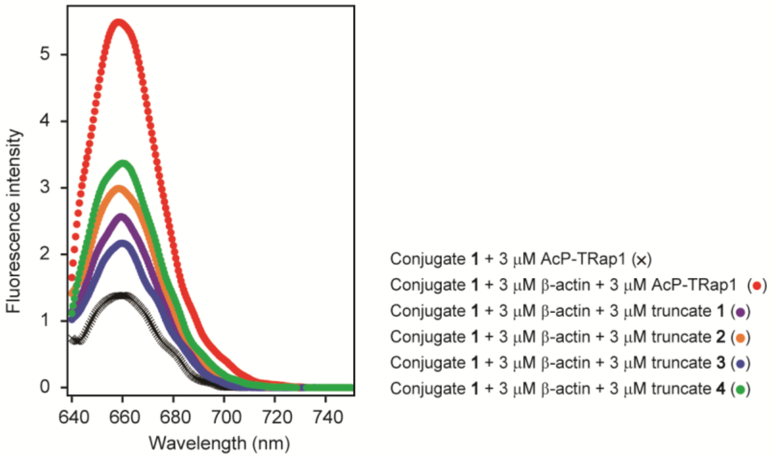

Supplementary Figure S5: Fluorescent response of truncated version of AcP-TRap1 upon binding with β-actin.

(**a**) Nucleotide sequences of AcP-TRap1 and its truncated versions. The conserved sequence of the AcP-TRap1 is shown in yellow. (**b**) Fluorescence intensities of conjugate **1** (final concentration: 1 μM) with the AcP-TRap1, truncate1, trancate2, trancate3, and trancate4 (3 μM) in the presence of a 3-μM β-actin (red, blue, purple, orange and green, respectively), and without AcP-TRap (black).

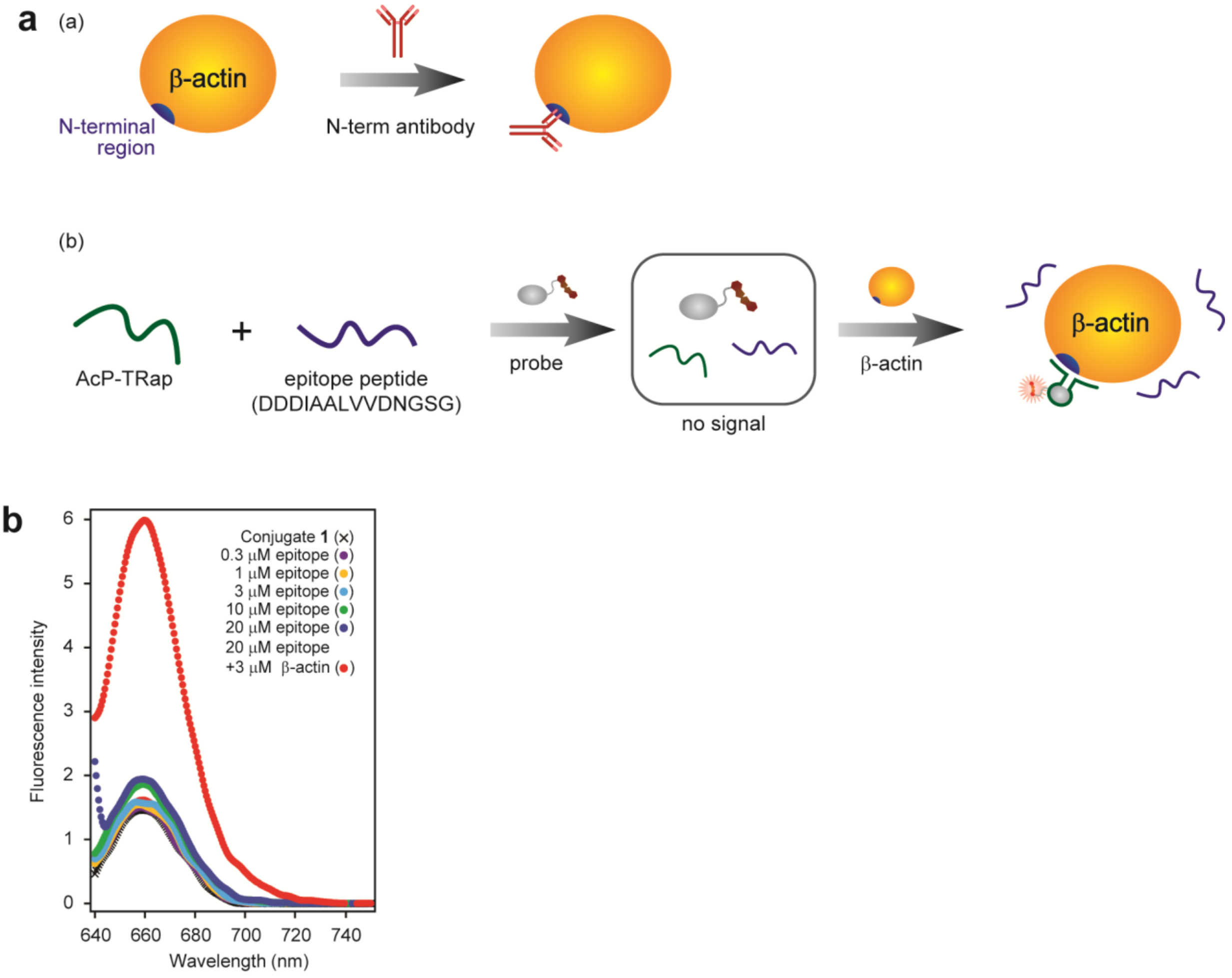

Supplementary Figure S6: Competitive binding analysis with epitope peptides.

(**a**) (a) Illustration of anti-β-actin antibody (BA3R) binding with β-actin. (b) Illustration of competitive binding of AcP-TRap1 to β-actin with an epitope peptide. (**b**) Fluorescence intensities of conjugate **1** (final concentration: 1 μM) with the AcP-TRap1 (3 μM) in the presence of 0.3- to 20-μM epitope peptides (purple, yellow, light blue, green and navy blue, respectively) and a 3-μM β-actin with a 20-μM epitope peptide. Remarkable binding of AcP-TRap1 with epitope peptide was not observed even in its highest concentration. Fluorescence intensity of conjugate **1** with AcP-TRap1 and without β-actin, is shown in black.

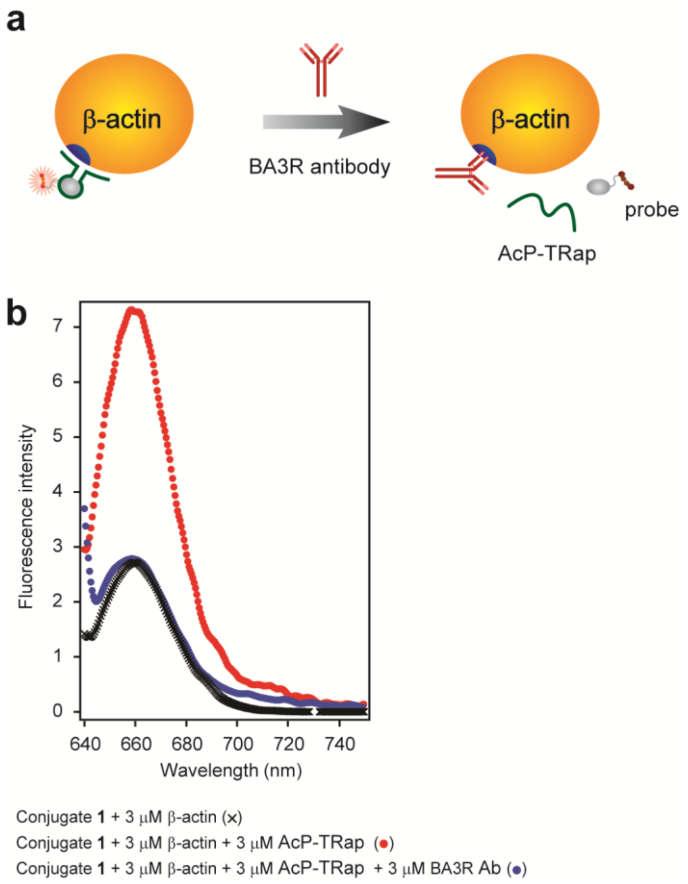

Supplementary Figure S7: Competitive binding analysis with an anti-β-actin antibody (BA3R).

(**a**) Illustration of competitive binding of AcP-TRap1 to β-actin with an anti-β-actin antibody (BA3R). (**b**) Fluorescence intensities of conjugate **1** (final concentration: 1 μM) with the AcP-TRap1 (3 μM) in the absence or presence of the anti-β-actin antibody (red and blue, respectively). Fluorescence intensity of conjugate **1** with β-actin and without AcP-TRap1, is shown in black.

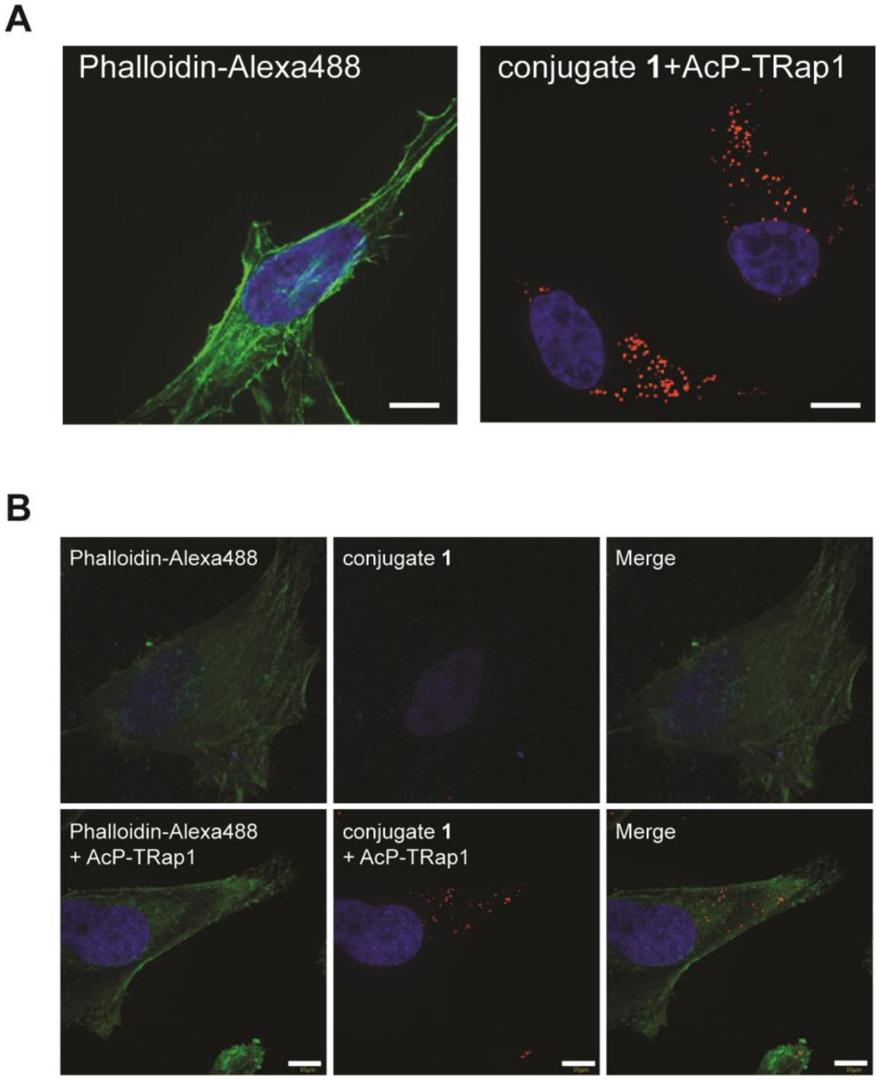

Supplementary Figure S8: Actin staining with AcP-TRap1 and phalloidin.
(**A**) Staining patterns of Phalloidin-Alexafluor-488 (green) and AcP-TRap1-conjugate **1** (red). (**B**) Co-localization experiments between Phalloidin-Alexafluor-488 (green) and AcP-TRap1-conjugate **1** (red). Scale bars are 10 μm. The nucleus is stained with Hoechst (blue).

a

BHQ1-binding stem-loop sequece

–UGGCCUAGAUAAAUUCGGAGCUU–

Clone	5' arm sequences	3' arm sequences
AcP-TRap1	GGAUCCAAGCUUGUUUGGCUCCAACCGUCCCCGCGUAUA	UUUGCCCAUCCUCAUAUAUUGCUUUCGACGGAGAAUUC
AcP-TRap1Ex	GGAUCCAAGCUUGUAUGGCUCCAACCGUCCCCGCGUAUA	AUUGCCCAUCCUCAUAUAUUGCUAUCGACGGAGAAUUC
AcP-TRap2	GGAUCCAAGCUUGUUUGGCAUUCCACGUCGAUAAAAGAU	AAAAUUCCCUCCUAUCUUUUGCUUUCGACGGAGAAUUC
AcP-TRap2Ex	GGAUCCAAGCUUGUAUGGCAUUCCACGUCGAUAAAAGAU	AAAAUUCCCUCCUAUCUUAUGCUAUCGACGGAGAAUUC

b

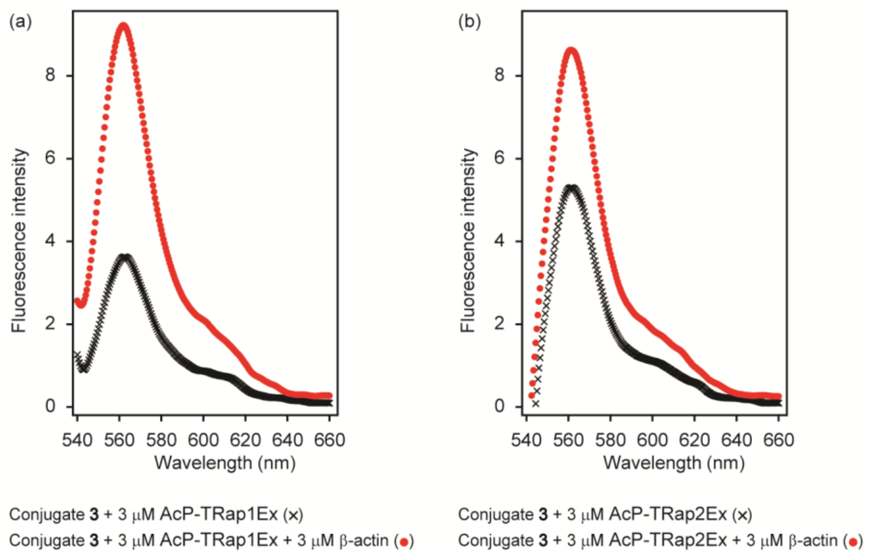

Supplementary Figure S9: Fluorescent response of expression version of AcP-TRap1 and AcP-TRap2 upon binding with β-actin.

(**a**) Nucleotide sequences of AcP-TRap1, AcP-TRap2, and their expression versions. The mutated nucleotides (U to A) are shown in yellow. (**b**) Fluorescence intensities of conjugate **3** (final concentration: 1 μM) with the AcP-TRap1Ex and AcP-TRap2Ex (3 μM) in the absence or presence of 3-μM β-actin (black and red, respectively).

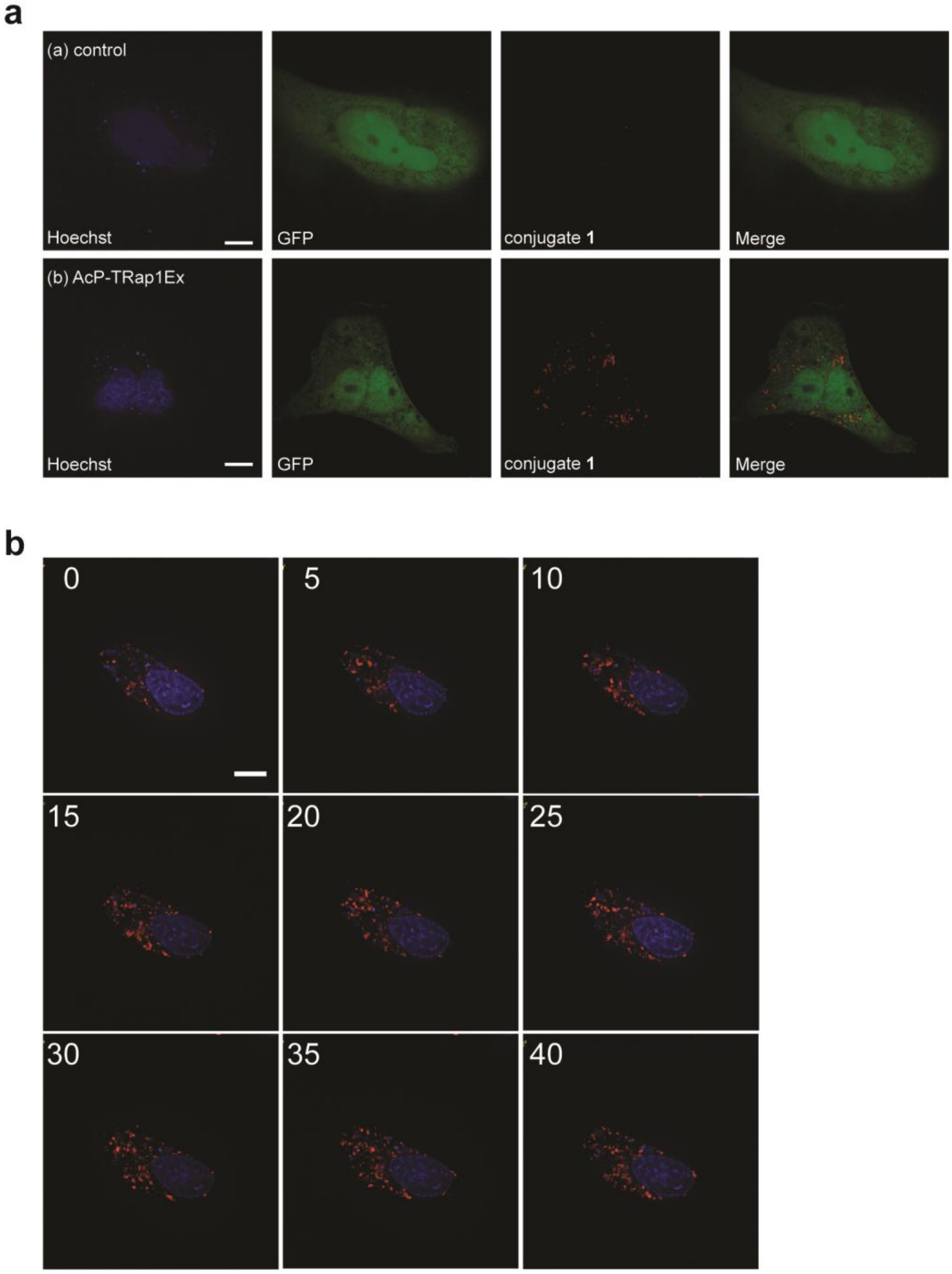

Supplementary Figure S10: Fluorescent response of expression version of AcP-TRap1 upon binding with β-actin.

(**a**) Visualization of β-actin in a single live HeLa cell with mock transfection control (a) or expressing AcP-TRap1Ex (b). Fluorescent images show Hoechst (blue), GFP (green), which was used as a marker for transfection, and conjugate **2** (red). (**b**) Time-lapse images of β-actin in a single live HeLa cell. The nucleus is stained with Hoechst (blue). All times are given in seconds. Scale bars, 10 μm.

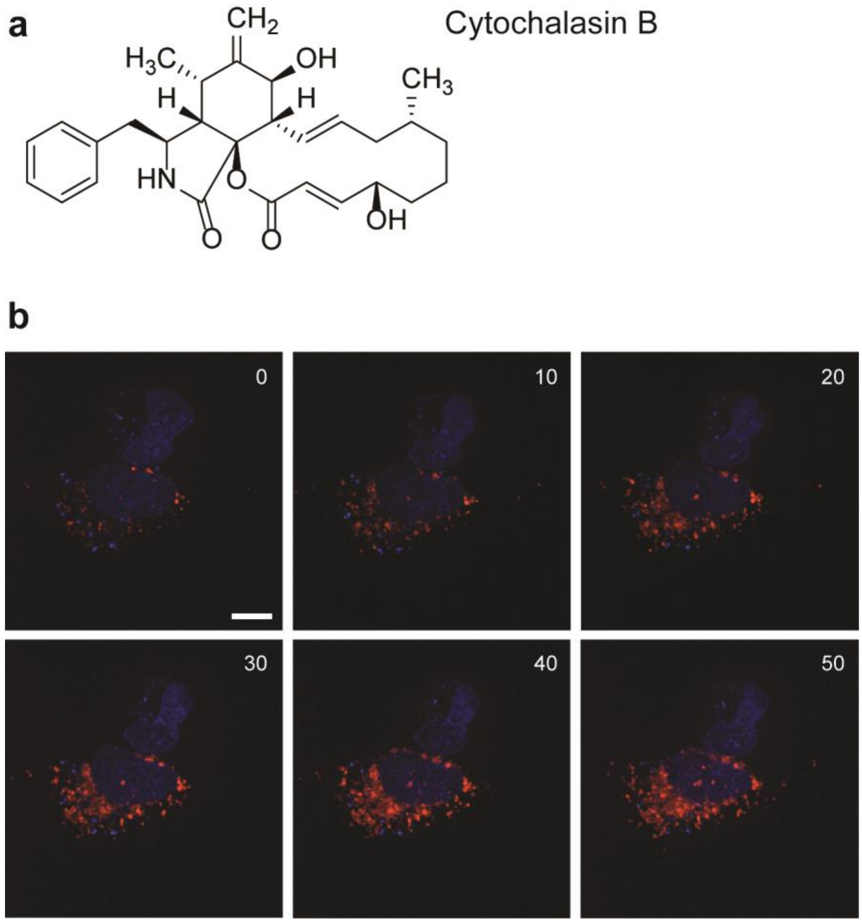

Supplementary Figure S11: Time-dependent increase of G-actin in a single HeLa cell after cytochalasin B treatment.

(**a**) Chemical structure of cytochalasin B. **(b)** Time-lapse images of G-actin in a single live HeLa cell after cytochalasin B treatment. The nucleus is stained with Hoechst (blue). All times are given in minutes. Scale bar, 10 μm.

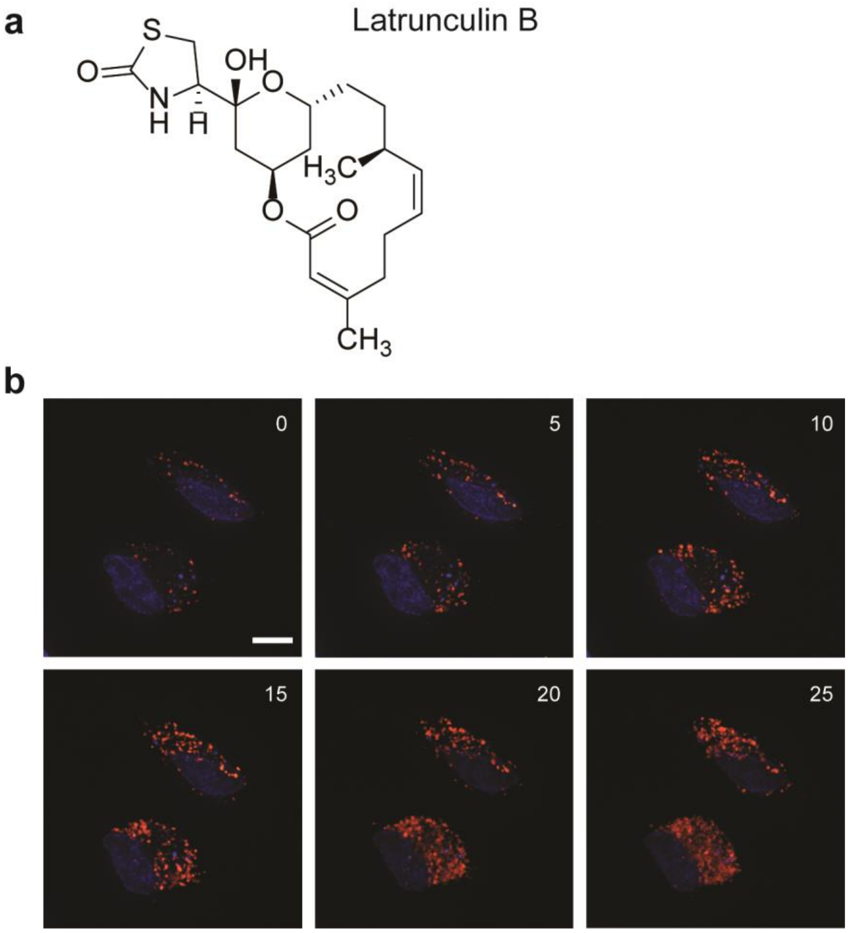

Supplementary Figure S12: Time-dependent increase of G-actin in HeLa cells after latrunculin B treatment.

(**a**) Chemical structure of latrunculin B. **(b)** Time-lapse images of G-actin in live HeLa cells after latrunculin B treatment. The nucleus is stained with Hoechst (blue). All times are given in minutes. Scale bar, 10 µm.

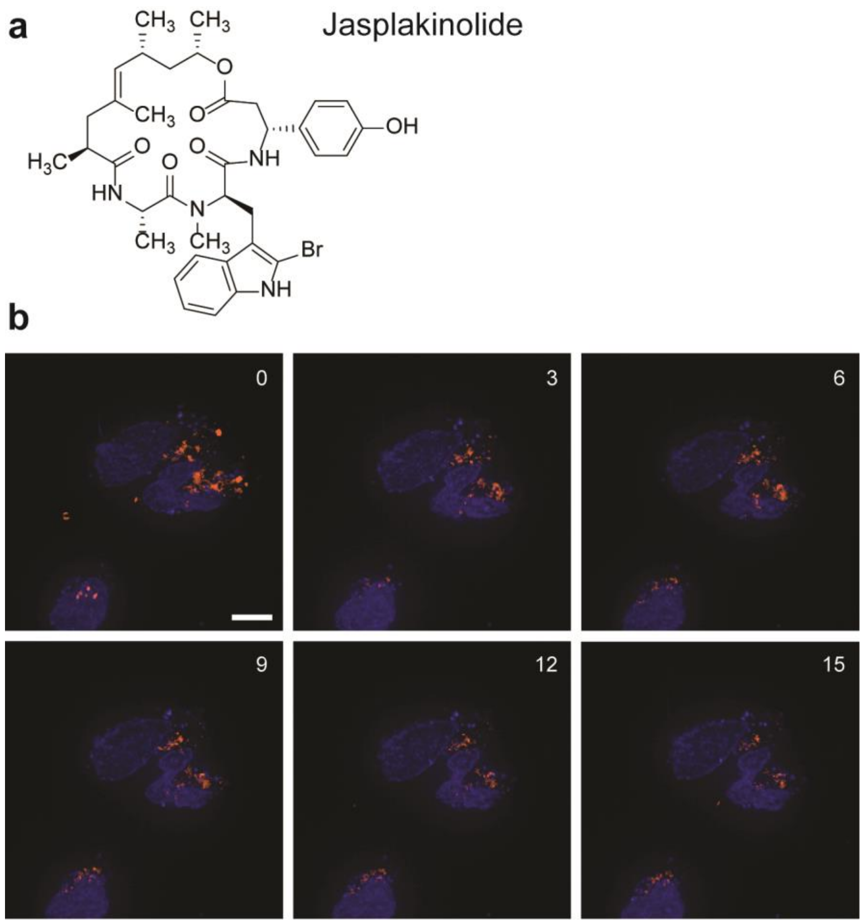

Supplementary Figure S13: Time-dependent decrease of G-actin in HeLa cells after jasplakinolide treatment.

(**a**) Chemical structure of jasplakinolide. **(b)** Time-lapse images of β-actin in live HeLa cells after jasplakinolide treatment. The nucleus is stained with Hoechst (blue). All times are given in minutes. Scale bar, 10 µm.

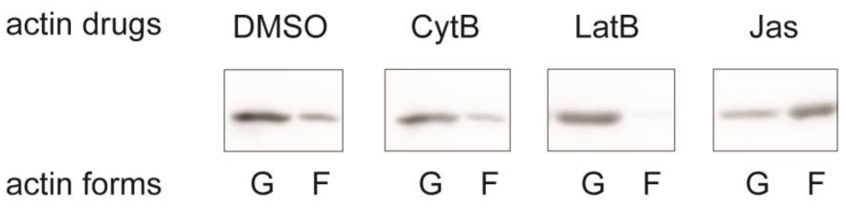

Supplementary Figure S14: Effects of actin drug treatment on the proportion of actin forms in cells.

Western blot analyses of actin in HeLa cells after actin-drug treatment. The methods for the analyses were in accordance with a previous report (supplementary ref.2). The concentrations of actin drugs were a 10-μM cytochalasin B (CytB), 1-μM latrunculin B (LatB), and 0.1-μM jasplakinolide (Jas), respectively. Treatment times were 30 minutes for LatB and Jas, and 90 minutes for CytB.

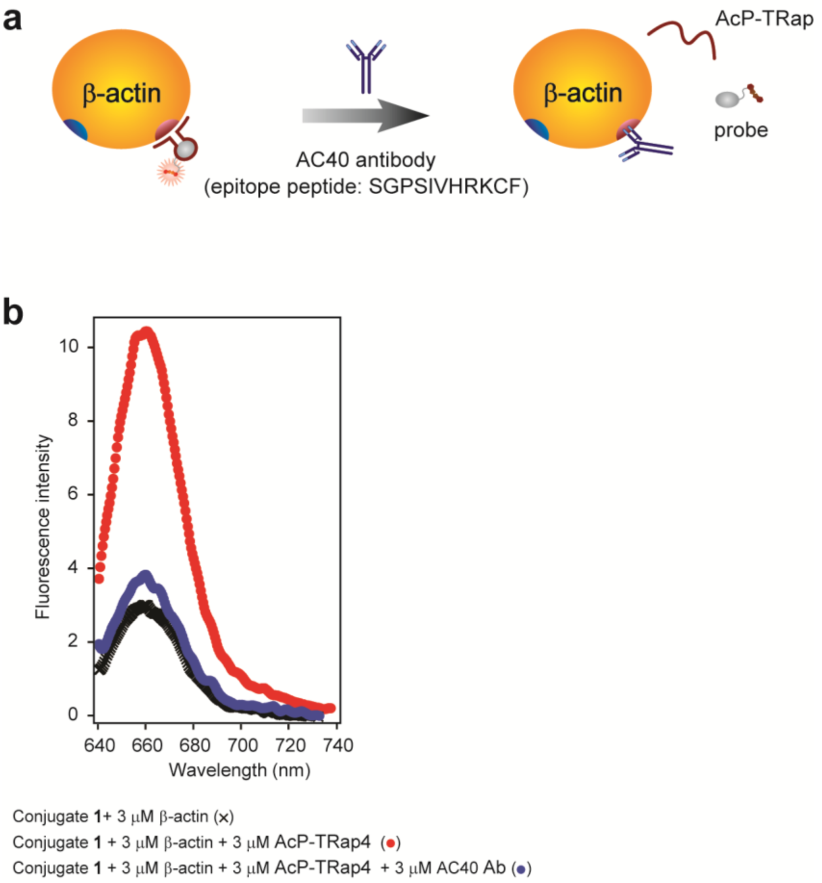

Supplementary Figure S15: Competitive binding analysis with an anti-β-actin antibody (AC40).

(**a**) Illustration of competitive binding of AcP-TRap4 to β-actin with an anti-β-actin antibody (AC40). (**b**) Fluorescence intensities of conjugate **1** (final concentration: 1 μM) with the AcP-TRap4 (3 μM) in the absence or presence of the anti-β-actin antibody (red and blue, respectively). Fluorescence intensity of conjugate **1** with AcP-TRap4 and without β-actin, is shown in black.

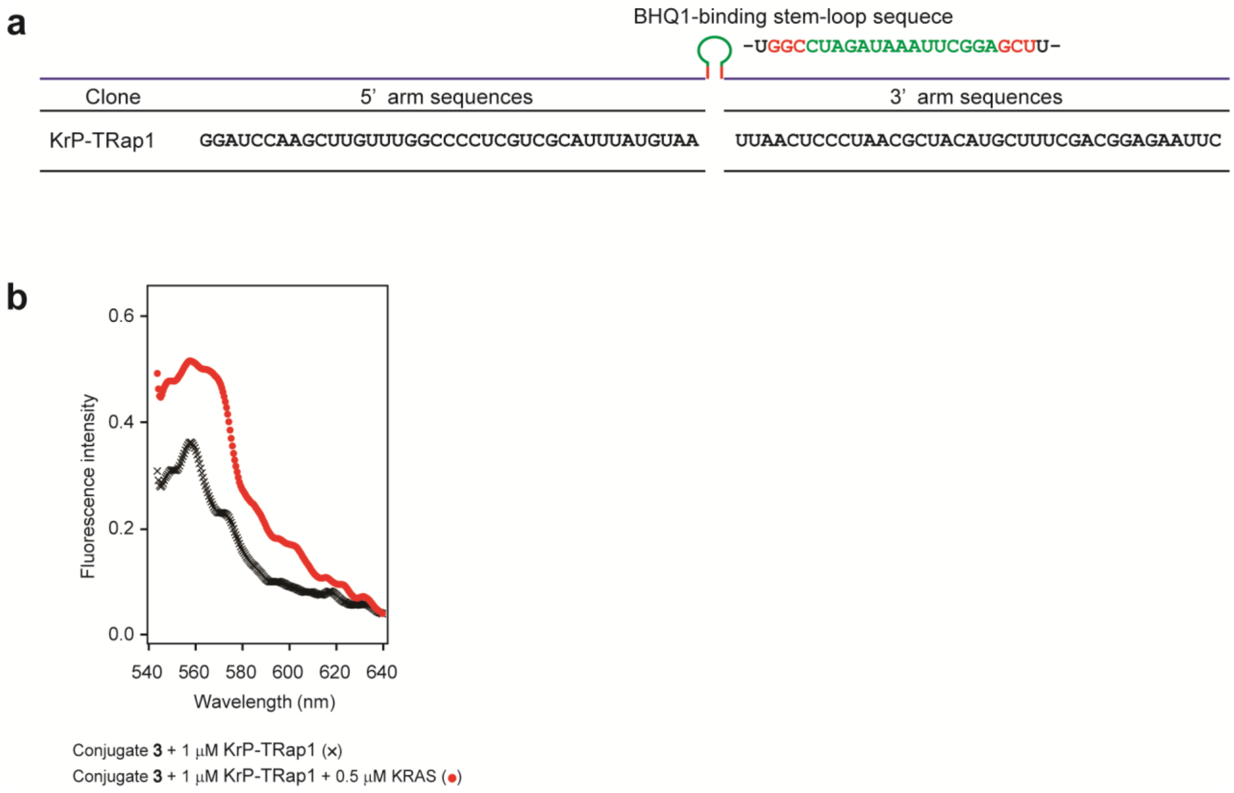

Supplementary Figure S16: Fluorescent response of KrP-TRap1 upon binding with KRAS. (**a**) Nucleotide sequences of KrP-TRap1. (**b**) Fluorescence intensities of conjugate **3** (final concentration: 1 µM) with the KrP-TRap1 in the absence or presence of 0.5-µM KRAS (black and red, respectively).

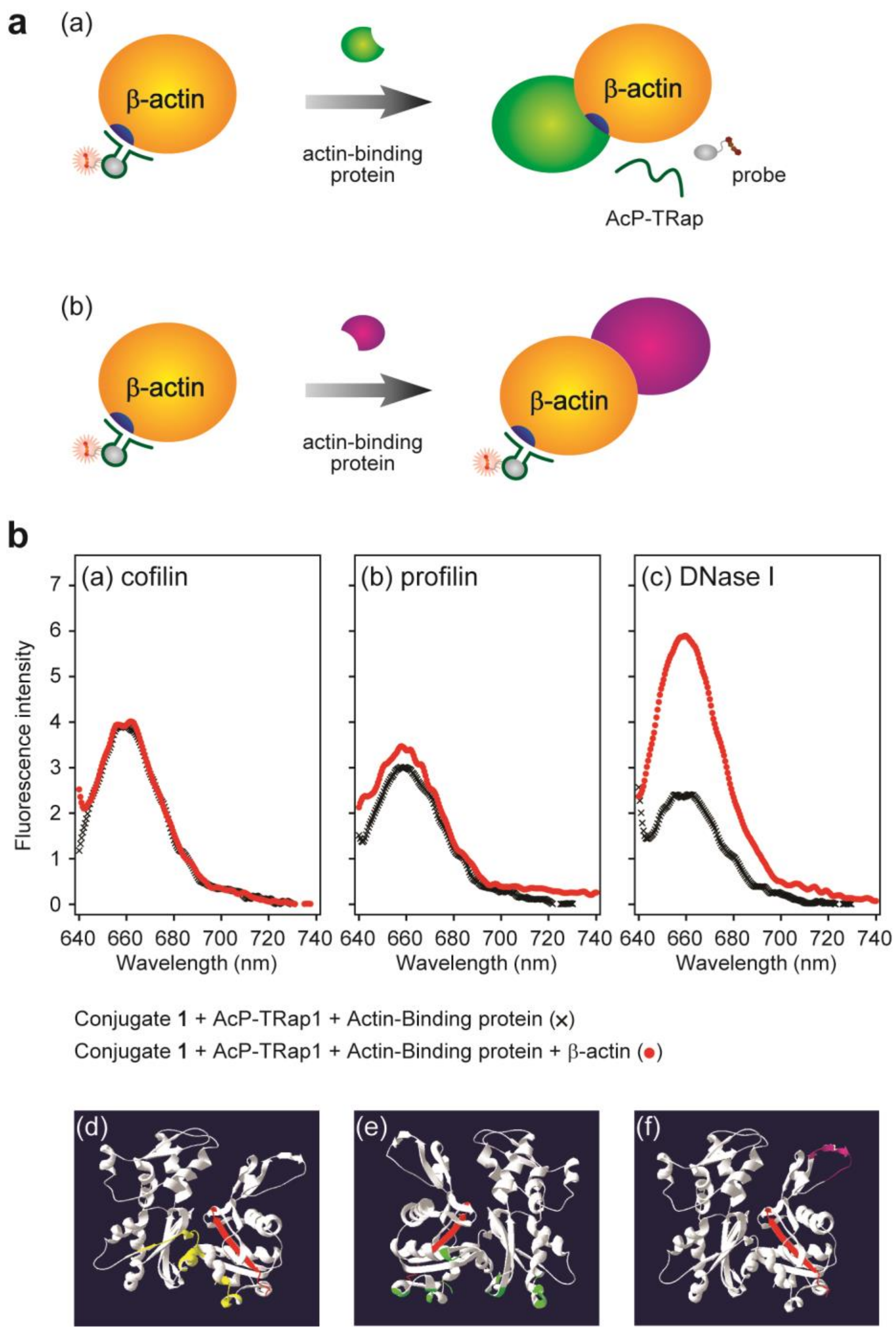

Supplementary Figure S17: Competitive binding analyses with actin-binding proteins.
(**a**) Illustration of competitive binding of AcP-TRap1 to β-actin with actin-binding proteins. One actin-binding protein competes with AcP-TRap1 (a) and another actin-binding protein dose not compete with AcP-TRap1 (b). **(b)** *In vitro* competition assays between AcP-TRap1 and cofilin 1 (a), profilin 1 (b), and DNase I (c). Fluorescence intensities of conjugate **1** (final concentration: 1 μM) with the AcP-TRap1 and β-actin in the absence or presence of 3-μM actin-binding proteins (black and red, respectively). Crystal structures at the bottom (PDB ID: 1ATN) highlight predicted binding surfaces for AcP-TRap1 (red), cofilin 1 ((d) yellow), profilin 1 ((e) green), and DNase I ((f) magenta).

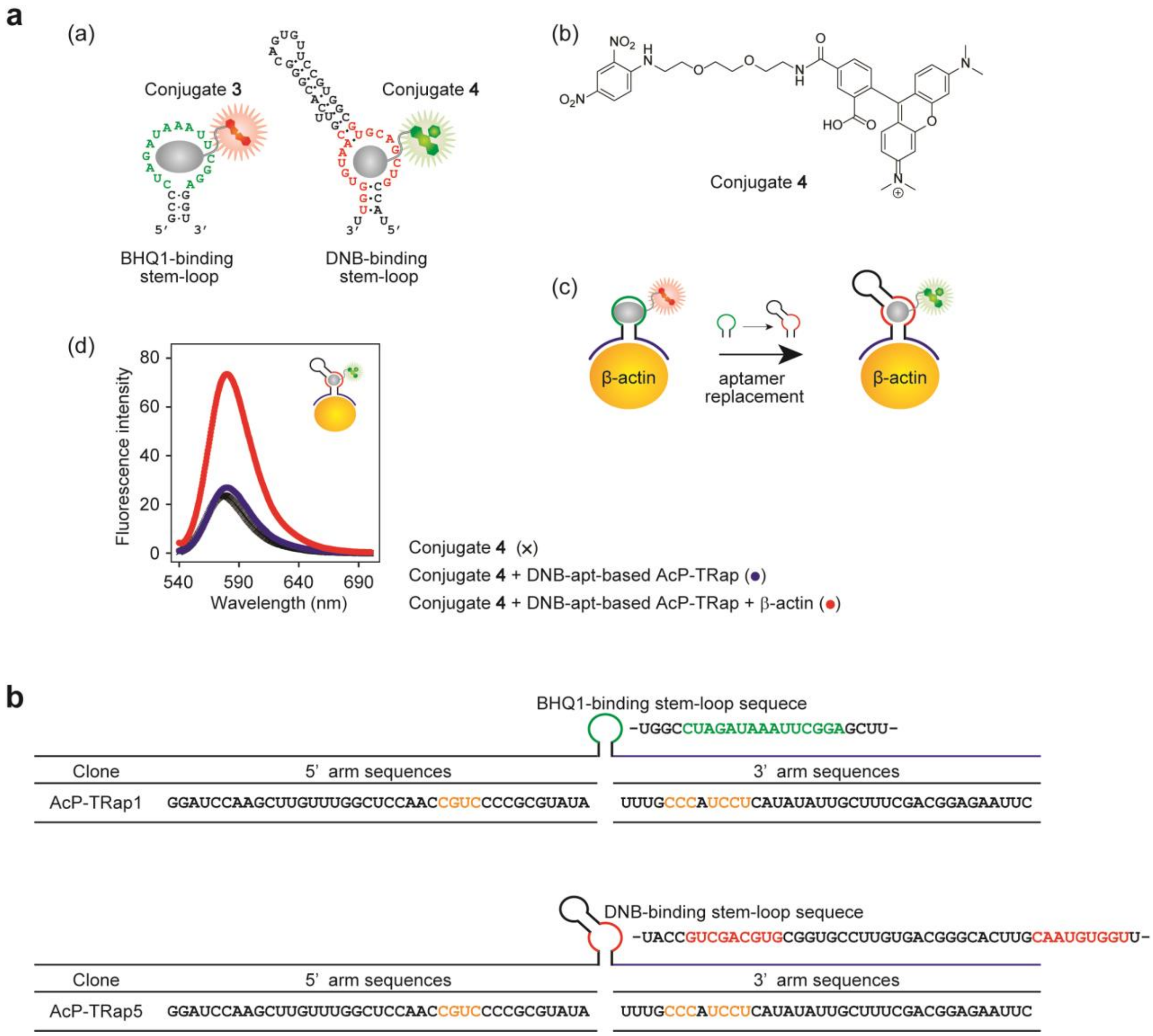

Supplementary Figure S18: Aptamer replacement of a BHQ1-binding stem-loop structure with a DNB-binding loop structure.

(**a**) (a) Secondary structure of RNA aptamers for fluorescent probes. (b) Chemical structure of conjugate **4**(supplementary ref.3). (c) Illustration of aptamer replacement of a BHQ1-binding stem-loop structure with a DNB-binding stem-loop structure. (d) Fluorescence intensities of conjugate **4** (final concentration: 2 μM) with the AcP-TRap5 (5 μM) in the presence or absence of a 5-μM β-actin (red and blue, respectively), and without AcP-TRap5 (black). **(b)** Nucleotide sequences of AcP-TRap1 and AcP-TRap5. The conserved sequences of the AcP-TRap are shown in yellow.

(a) in vitro selection with BA3R

BHQ1-binding stem-loop sequece

-UGGCCUAGAUAAAUUCGGAGCUU-

Clone	5' arm sequences	3' arm sequences	clone number
AcP-TRap1	GGAUCCAAGCUUGUUUGGCUCCAACCGUCCCCGCGUAUA	UUUGCCCAUCCUCAUAUAUUGCUUUCGACGGAGAAUUC	2
AcP-TRap2	GGAUCCAAGCUUGUUUGGCAUUCCACGUCGAUAAAAGAU	AAAAUUCCCUCCUAUCUUUUGCUUUCGACGGAGAAUUC	3
AcP-TRap3	GGAUCCAAGCUUGUUUGGCCCAAUAACGUCGAUUGAUUG	UCAAUGAAUUACACAACAAUGCUUUCGACGGAGAAUUC	1
	GGAUCCAAGCUUGUUUGGCCUAUUGCGUCGGAGAUCAGC	AAUUGCCCAUCCUGCUGUUCGCUUUCGACGGAGAAUUC	8
	GGAUCCAAGCUUGUUUGGCCCACCGUCCUAAUGGAGUUU	CAUCCUUCCUCAAAACUCAAGCUUUCGACGGAGAAUUC	1
	GGAUCCAAGCUUGUUUGGCAUUCUCACUUAACUAUGUAA	AAAAUUCCCUCCUAUCUUUUGCUUUCGACGGAGAAUUC	1
	GGAUCCAAGCUUGUUUGGCCCCAUUCACAGACGGCGUAU	UUACAUCUUCACACCUAACCGCUUUCGACGGAGAAUUC	1
	GGAUCCAAGCUUGUUUGGCCUACGUCAACCUCAUUAUUG	CAGCCCUCCUGCAAUUUUGAGCUUUCGACGGAGAAUUC	1
	GGAUCCAAGCUUGUUUGGCUUCCAUCCAACUGUAAUCAC	UGAUUGCGCCGUCACCUUAUGCUUUCGACGGAGAAUUC	1
	GGAUCCAAGCUUGUUUGGCCCAGUCGGACCUAUCUUGUG	UUGCCCAUCCUCACAAGUUCGCUUUCGACGGAGAAUUC	1
	GGAUCCAAGCUUGUUUGGCUCUCAGCGUCGAACGUUUUA	ACGCCCAUUCCUAUGAAUACGCUUUCGACGGAGAAUUC	2

(b) in vitro selection with AC40

BHQ1-binding stem-loop sequece

-UGGCCUAGAUAAAUUCGGAGCUU-

Clone	5' arm sequences	3' arm sequences	clone number
AcP-TRap4	GGAUCCAAGCUUGUUUGGCCCAAUUCCUCGUCUUCAGAC	GUUCACAACCUCUACUUCCUGCUUUCGACGGAGAAUUC	4
	GGAUCCAAGCUUGUUUGGCCUACCCAGUCGAUCUCGUCA	UUAGUUUCCCGAUCCUUGACGCUUUCGACGGAGAAUUC	2
	GGAUCCAAGCUUGUUUGGCCCUUACGCGUCGAACGCCUC	UUACCCGGUCCUCUGCUAUGGCUUUCGACGGAGAAUUC	1
	GGAUCCAAGCUUGUUUGGCCCUUUCCUUUCUUCGGCGUU	GUUCUUUUAUAGCCGCUUGUGCUUUCGACGGAGAAUUC	1
	GGAUCCAAGCUUGUUUGGCCCUUUUCUUUCCCCGCCGUU	UUUUUAGCUGUUACUUUGACGCUUUCGACGGAGAAUUC	1
	GGAUCCAAGCUUGUUUGGCCCUUACGUUGACUGCUACCU	AAAUCUUCCUACUACCUGGUGCUUUCGACGGAGAAUUC	1
	GGAUCCAAGCUUGUUUGGCCGCCCUUUUCCGUUGCCUGU	UUUCUAAAAAUAGCGCUCUUGCUUUCGACGGAGAAUUC	1
	GGAUCCAAGCUUGUUUGGCCCUUUUGUCGAACCCGCGGG	UAUGUUCCCAACACUCGCGUGCUUUCGACGGAGAAUUC	1

Supplementary Figure S19: Nucleotide sequences of AcP-TRaps.

Nucleotide sequences of selected RNAs with an anti-β-actin antibody clone BA3R (a) or clone AC40 (b) are shown. The conserved sequences of the AcP-TRap are shown in yellow.

Supplementary Videos

Supplementary Video S1-S2.

Time-lapse images of G-actin and the nucleus (blue) in live cells. Images were taken every 5 minutes for 1 hr (**Supplementary Video S1**) and 1.5 minutes for 10.5 min (**Supplementary Video S2**), and playback is at 3 frames per second.

Supplementary Video S3-S4.

Time-lapse images of G-actin (red) and the nucleus (blue) (**Supplementary Video S3**), and with the GFP expression as a transfection control (Green) (**Supplementary Video S4**) in a single live HeLa cell after cytochalasin B treatment. The area of images is the same as in **Fig. 4b**. Images were taken every 10 minutes for 2 hrs, and playback is at 3 frames per second.

Supplementary Video S5-S6.

Time-lapse images of G-actin (red) and the nucleus (blue) in live HeLa cells after latrunculin B treatment. Images were taken every 5 minutes for 1 hr, and playback is at 3 frames per second.

Supplementary Video S7-S8.

Time-lapse images of G-actin (red) and the nucleus (blue) in live HeLa cells after jasplakinolide treatment. Images were taken every 3 minutes for 30 minutes, and playback is at 3 frames per second.

Supplementary References

1. Sato, S., *et al. Angew. Chem. Int. Ed.* **54**, 1855-1858 (2015).
2. Yatsuzuka, K., *et al. Chem. Commun.* **54(52)**, 7151-7154 (2018).
3. Lockett, S., *et al. Cytometry A.* **85(6)**, 512-521 (2014).
